# Comparative transcriptomic and proteomic analyses of hypoxia response in wild and cultivated tomato roots

**DOI:** 10.1186/s12864-025-11653-3

**Published:** 2025-06-02

**Authors:** Zhihan Zhang, Yabing Hou, Hao Yin, Song Lu, Daliang Liu, Lin Cheng, Houlin Yu, Tao Li, Yiyong Zhao

**Affiliations:** 1 Present Address: Key Laboratory of Functional Agriculture in Higher Education Institutions of Guizhou Province, College of Agriculture, Guizhou University, Guiyang, 550025 China; 2 State Key Laboratory of Public Big Data, College of Computer Science and Technology, Guizhou University, Guiyang, 550025 China; 3 College of Horticulture, Nanjing Agricultural University, Nanjing, 210095 China; 4 Henan International Joint Laboratory of Tea-Oil Tree Biology and High Value Utilization, Xinyang Normal University, Xinyang, 464000 China; 5 Department of Biochemistry and Molecular Biology, University of Massachusetts Amherst, Amherst, MA 01003 USA

**Keywords:** Tomato, Root, Hypoxia, RNA-seq, Proteome, Positive selection, Protein-protein interaction

## Abstract

**Background:**

Hypoxia significantly impairs tomato productivity and yield. Although cultivated tomato varieties (*Solanum lycopersicum*) are generally sensitive to low-oxygen conditions, their wild relatives (*Solanum habrochaites*) display substantially lower sensitivity. To elucidate the molecular mechanisms underlying these contrasting phenotypes, as well as the impact of positive selection and protein-protein interactions of differentially expressed genes (DEGs) and proteins (DEPs), we conducted transcriptomic and proteomic analyses of root samples from a wild tomato accession, T178 (*S. habrochaites*), and a cultivated tomato variety, Fenzhenzhu (*S. lycopersicum*, FZZ).

**Results:**

Compared with cultivated seedlings, wild tomato seedlings exhibited markedly reduced sensitivity to hypoxia, as demonstrated by lower growth inhibition and higher membership function values under low-oxygen conditions. In T178, 2,351 DEGs were identified (1,238 upregulated and 1,113 downregulated), whereas in FZZ, 2,931 DEGs were detected (1,326 upregulated and 1,605 downregulated). Heatmap clustering and functional enrichment analysis revealed significant differences in transcriptional regulation between T178 and FZZ under hypoxia. Specifically, among the unique DEGs in T178, genes related to carbohydrate metabolism were significantly upregulated, whereas genes associated with single-organism metabolic processes were downregulated. In contrast, among the unique DEGs in FZZ, genes related to DNA-templated transcription were significantly upregulated, whereas genes associated with protein phosphorylation were downregulated. Proteomic analysis identified 544 and 493 DEPs in T178 and FZZ, respectively, with T178 DEPs predominantly linked to metabolic flexibility and antioxidant responses, whereas both sets were enriched in similar metabolic pathways. Further positive selection analyses emphasized the adaptive evolution of hypoxic responses in wild and cultivated tomatoes, exemplified by T178, which harbors 1,289 positively selected genes linked to carbon metabolism and energy homeostasis, underscoring its adaptation to low-oxygen environments. Moreover, protein-protein interaction (PPI) network analyses revealed distinct adaptive strategies in T178 and FZZ. By analyzing the gene and protein networks of FZZ and T178 under hypoxic conditions, we inferred that T178 enhances hypoxia adaptation by forming more independent small modules and multilevel regulatory networks, whereas FZZ relies on a few large modules with limited functional diversity, resulting in weaker hypoxia tolerance.

**Conclusions:**

Our results demonstrated that the molecular response mechanisms to hypoxia differ substantially between wild and cultivated tomatoes, with wild tomatoes showing more distinctive and effective adaptations. The differentially regulated genes identified in this study represent promising targets for future research and breeding efforts aimed at improving hypoxia tolerance in tomatoes.

**Supplementary Information:**

The online version contains supplementary material available at 10.1186/s12864-025-11653-3.

## Background

Oxygen is an essential prerequisite for normal physiological metabolism and the growth and development of higher plants. Moreover, growth, development, and metabolism of living plant cells are intricately linked to oxygen availability. Hypoxia severely affects plant growth, development, and physiological metabolism. Waterlogging can be defined as excess water in the soil [1]. Waterlogging conditions result in a low oxygen (hypoxia) environment in the rhizosphere due to the sluggish diffusion of oxygen in water [1, 2]. Waterlogging poses a significant obstacle to the optimal growth of plants. It is estimated that approximately 16% of the earth’s arable land is adversely affected by soil waterlogging. The consequences of soil waterlogging are profound, leading to substantial reductions in crop yields, with potential losses of up to 80% [3]. This widespread issue has caused significant damage to the agricultural output worldwide.

Under waterlogged conditions, roots serve as the primary organ responsible for sensing hypoxia, which refers to a reduced oxygen environment. This hypoxic condition hampers the supply of oxygen to the root system [4, 5]. Hypoxia also disrupts energy production and cellular homeostasis through various mechanisms, including the inhibition of mitochondrial respiration, alterations in the redox state, the accumulation of reactive oxygen species (ROS), and the occurrence of calcium ion (Ca^2+^) spiking [6–8]. Furthermore, previous studies have demonstrated that hypoxia stress can disrupt root porosity and impede oxygen transport in species that are particularly sensitive to hypoxic conditions [9]. Nevertheless, the development of adaptive traits, including the formation of adventitious roots, aerenchyma formation, and increased root porosity, has been identified as key factors contributing to enhanced plant tolerance to hypoxic conditions [6, 7, 10].

Plants respond to hypoxic conditions through morphological and physiological adaptations that are tightly regulated by the expression of relevant genes and subsequent protein synthesis [11]. Furthermore, hypoxia can result in drastic changes in transcription, translation, and metabolite levels in *Arabidopsis* [12–14], rice [15], maize [16], and cotton [17]. RNA-seq analysis has been widely used to elucidate the underlying molecular responses associated with plant stress resistance, as well as the complex signaling pathways involved in crosstalk between different stress response mechanisms [18, 19]. Studies have identified a set of hypoxia-induced genes in *Arabidopsis thaliana* that encode various metabolic activities, including those involved in glycolysis and fermentation [14]. Furthermore, hypoxia significantly alters the transcript levels of genes related to these pathways as well as ethylene synthesis and perception, calcium signaling, and nitrogen utilization [20]. In maize, the expression of genes regulating energy production, aerenchyma formation, and ethylene signaling changes under hypoxia conditions [16]. Recently, comparative transcriptomic analysis has shed light on the molecular responses of lowland and upland rice to hypoxic conditions. Notably, this analysis has revealed the presence of exclusive differentially expressed genes that are particularly enriched in the pathways associated with plant hormone metabolism and signaling [5].

As one of the most important horticultural crops in the world, tomatoes are deeply loved by consumers. However, similar to other plants, normal physiological processes are affected by hypoxia. For example, studies have shown that the duration of hypoxia has a significant impact on gene expression in tomatoes. Specifically, under short-term hypoxic conditions, 267 genes in the roots of the tomato variety *Moneymaker* showed changes in expression, whereas under long-term hypoxia, this number increased to 1,421, with 243 genes exhibiting changes in expression under both conditions [21].

Moreover, compared to plants with well-developed aerenchyma, such as rice, tomatoes display distinct responses to hypoxia. Rice possesses an efficient system for long-distance oxygen transport, facilitated by aerenchyma formation, which allows oxygen to diffuse from the shoots to the roots and supports root respiration, even under waterlogged conditions. This adaptation is particularly effective in flood-prone environments, as it minimizes radial oxygen loss and maintains aerobic conditions in the root zone [22]. In contrast, tomatoes lack extensive aerenchyma development and rely on metabolic adjustments to cope with hypoxia. Proteomic studies have revealed that hypoxia-sensitive and hypoxia-tolerant tomato accessions exhibit significant differences in the expression of proteins related to glycolysis, fermentation, and amino acid metabolism, with hypoxia-tolerant accessions upregulating proteins involved in anaerobic respiration and antioxidant defense and downregulating those associated with aerobic metabolism [23]. Furthermore, the study identified candidate proteins associated with stress priming, suggesting that pre-exposure to mild hypoxia can enhance the tolerance of tomato roots to subsequent severe hypoxia [23]. This metabolic reprogramming highlights the importance of alternative pathways in tomato hypoxia tolerance and provides a basis for further investigation of the molecular mechanisms underlying these responses.

Selection pressure, also known as natural selection pressure, refers to environmental or internal factors that affect the survival and reproductive success of a species. These pressures do not directly cause genetic changes but instead act on existing genetic variation within a population, favoring individuals with traits that confer higher adaptability. Over time, this process can lead to changes in the frequency of certain alleles within a population, thereby driving the evolution of the species [24]. Genetic variation, which is the raw material for natural selection, arises through neutral processes, such as mutation and genetic drift, as described by the neutral theory of molecular evolution. This variation is not inherently adaptive but provides the diversity upon which natural selection can act. For example, studies have shown that *Solanum cheesmaniae* and *Solanum galapagense* grow on the Galápagos Islands. These species are frequently exposed to seawater and exhibit significant natural variations in their responses to high-salinity stress [25]. This variation likely arose through neutral processes, but the selective pressure of high salinity favored individuals with greater salt tolerance, allowing these wild tomato species to thrive in their environment. In contrast, cultivated tomato, which was domesticated from wild tomato species [21], is highly sensitive to flood stress [26, 27]. With the development of facility agriculture and the improvement of cultivation techniques, nutrient solution cultivation has been widely adopted in tomato cultivation. However, tomato is usually susceptible to rhizosphere hypoxia during hydroponic nutrient solution cultivation. It has been noted that there are some differences between wild and cultivated tomatoes in terms of their resistance to abiotic stresses [28]. For example, wild tomato varieties are more cold-tolerant than cultivated tomatoes [29], and compared to cultivated tomatoes, wild tomato varieties exhibit lower sensitivity to hypoxia [30].

In this study, we performed transcriptome analyses to elucidate the molecular mechanism underlying the greater tolerance of wild tomato to hypoxia stress than that of cultivated tomato. RNA-seq analysis was used to investigate the different expression profiles of hypoxia-treated roots in wild tomato T178 and cultivated tomato FZZ. We uncovered the potential functional pathways and regulatory genes differentially expressed in T178 and FZZ. These results offer the gene resources to be further used by breeders and scientists for developing tolerant tomato genotypes to improve their productivity under hypoxia.

## Methods

### Cultivation and treatment of tomato seedlings

Seeds of the wild tomato *S. habrochaites* T178 (Accession No. LA1777) were obtained from the Tomato Genetics Resource Center (TGRC), and seeds of the cultivated tomato *S. lycopersicum* ‘Fenzhenzhu’ were provided by Henan Yuyi Seed Industry Technology Co., Ltd. (Zhengzhou, China). Germinated tomato seeds were transplanted to the solid medium (vermiculite: peat: perlite = 2: 1: 1). Consistent and healthy seedlings were selected and transferred to hydroponic conditions. All plants were cultured with a modified Hoagland nutrient solution containing: 3.50 mmol/L Ca(NO_3_)_2_, 5.00 mmol/L KNO_3_, 1.00 mmol/L KH_2_PO_4_, 2.00 mmol/L MgSO_4_, 46.00 µmol/L H_3_BO_3_, 6.70 mmol/L MnCl_2_, 0.32 mmol/L CuSO_4_, 0.76 mmol/L ZnSO_4_, 0.11 mmol/L Na_2_MoO_4_, and 50.00 µmol/L EDTA-Fe. The pH was adjusted to 6.5, as described in the previous report [31]. Cultivation of all seedlings was carried out in a greenhouse, maintaining the temperature conditions at 28 °C (day) and 25 °C (night), respectively. The photoperiod was set to a 14 h light/10 h dark cycle with a light intensity of 500 µmol m^− 2^ s^− 1^ to simulate natural growth conditions.

The nutrient solution was replenished three times per week. Four-week-old seedlings were subjected to 12-day immersion in solutions containing varying concentrations of dissolved oxygen. In the aerated treatment (A), fresh air was infused into the solution, maintaining the dissolved oxygen concentration within a range of 7.0–8.0 mg/L. On the other hand, a hypoxic environment (N) was established by introducing nitrogen gas into the solution, subsequently reducing the dissolved oxygen concentration to between 0.5 and 2.0 mg/L. Concentrations of dissolved oxygen were regulated via a digital monitoring system. This system was designed to provide real-time tracking of fluctuations in the dissolved oxygen concentration, enabling automatic adjustments when necessary.

**Fig. 1:**
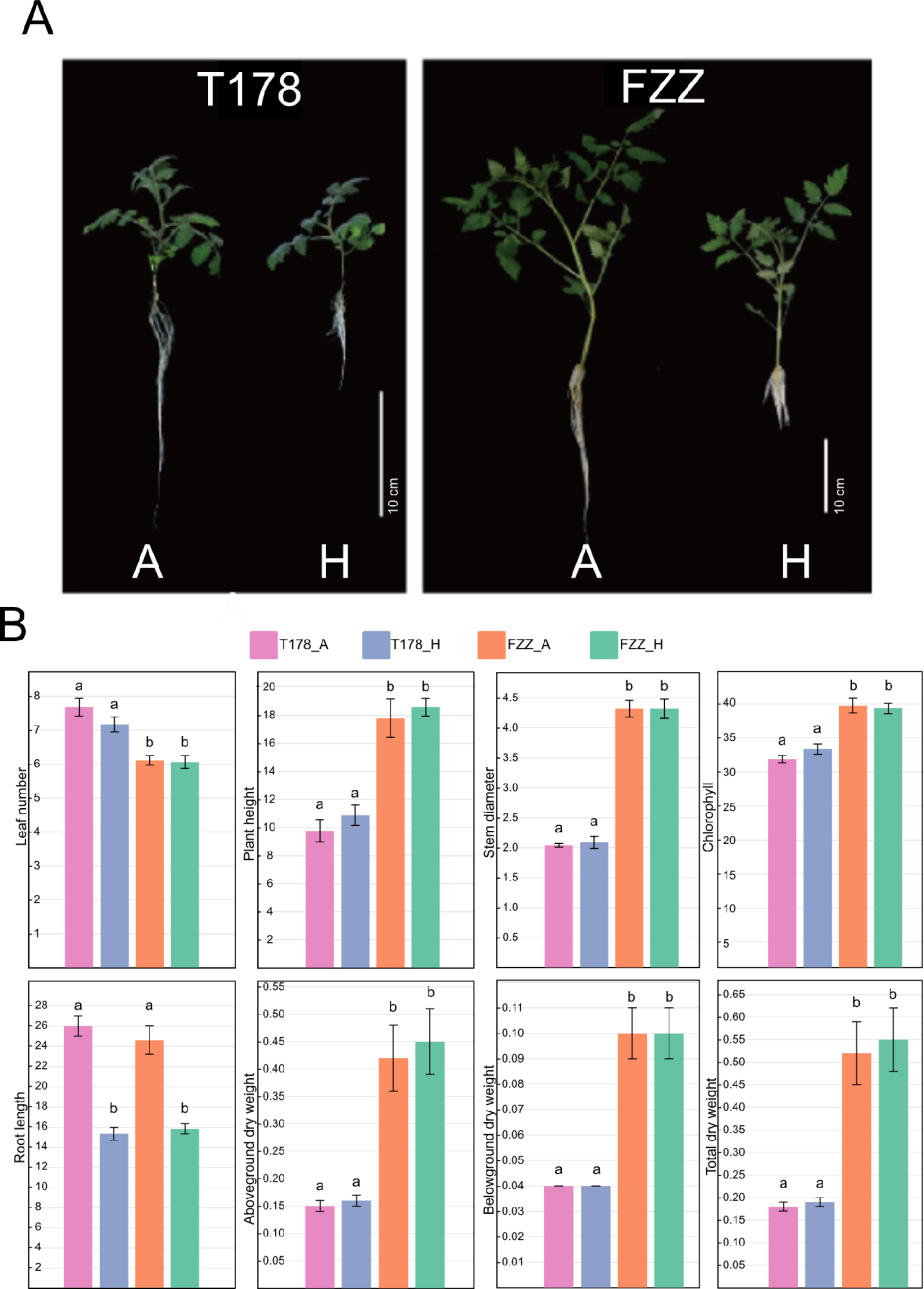
Effects of oxygen availability on growth and dry weight of tomato seedlings. (**A**) Representative images of wild tomato (T178) and cultivated tomato (FZZ) seedlings grown under aerated (**A**) and hypoxic (N) conditions for 12 days. Scale bar: 10 cm. (**B**) Quantitative analysis of eight growth traits in T178 and FZZ seedlings under aerated and hypoxic conditions. After 12 days, 12 seedlings from each treatment group were randomly selected to measure leaf number, plant height (cm), stem diameter (mm), chlorophyll content (SPAD), root length (cm), aboveground dry weight (g), belowground dry weight (g), and total dry weight (g). Data are presented as the mean ± standard error. Significant differences among groups were determined using Tukey’s honestly significant difference (HSD) test at *P* < 0.05. Bars labeled with different letters (a, b) indicate that those groups differed significantly from each other, whereas bars sharing the same letter were not significantly different

After 12 days of hydroponic growth, 12 tomato seedlings were randomly selected from each treatment group. The plant samples were blanched at 105 °C for 15 min and subsequently dried at 80 °C until a constant weight was achieved. The total dry weight was then precisely measured using an analytical balance with a resolution of one ten-thousandth of a gram (Fig. 1).

### Transcriptomic sequencing and analysis

After 12 days of hypoxic treatment, root samples were harvested, immediately frozen in liquid nitrogen, and then stored at -80 °C to facilitate total RNA extraction and cDNA library construction. RNA was extracted from two independent biological replicates, using Trizol (Invitrogen) according to the manufacturer’s instructions. The concentration and purity of the extracted RNA were determined spectrophotometrically using a NanoDrop 2000 spectrophotometer (Germany) and an Agilent 2100 Bioanalyzer (Agilent Technologies, Palo Alto, CA, USA). Then the cDNA libraries were constructed according to the previous study [32], and the sequencing was carried out on the BGISEQ-500 platform at BGI Genomics Co., Ltd., (Shenzhen, China), and 2 × 100 bp paired-end reads were generated.

### Identification of differentially expressed genes for RNA-seqs analyses

The raw sequencing reads underwent a pre-processed procedure, utilizing the SOAPnuke [33]. Subsequent filtration was performed with Trimmomatic [34] to eliminate adaptor sequences, reads possessing an unknown nucleotide (‘N’) composition exceeding 5%, and low-quality reads. Following these procedural steps, clean reads were successfully isolated and archived in the FASTQ format. For the cultivated tomato FZZ, the reference genome used was *S. Lycopersicum* (accessible at https://data.jgi.doe.gov/refine-download/phytozome?organism=Slycopersicum), whereas for the wild tomato T178, the reference genome was *S. habrochaites* (accessible at https://ngdc.cncb.ac.cn/gwh/Assembly/25902/show). The clean reads were initially mapped onto the reference genome sequences using HISAT2 (v2.2.1) [35]. Subsequent quantification of gene expression was facilitated by StringTie (v2.2.1) [36]. Differentially expressed genes (DEGs) between the hypoxic and aerated treatments were identified through DESeq2 [37]. The statistical test used to determine the significance of expression differences calculates the P-value based on a negative binomial distribution. Criteria for designation as DEGs included a P-value below 0.05 and an absolute log2FoldChange greater than one. The gene IDs of the two species were unified using a reciprocal best-hit strategy for identifying orthologs through the BLASTP program of the BLAST software suite (v2.2.28) [38]. A subsequent Venn diagram analysis was performed, and the DEGs were subjected to enrichment analysis, including Gene Ontology (GO) and Kyoto Encyclopedia of Genes and Genomes (KEGG) analyses, with an FDR value of less than 0.05.

### Proteomic analysis: protein extraction, identification, and quantification

This study utilized the same samples as described for the generation of transcriptome data, where protein extraction, iTRAQ labeling, and quantification were performed by BGI Genomics Co., Ltd. (Shenzhen, China). Initially, the proteins were extracted using iTRAQ reagents and quantified via the Bradford method, followed by the detection through Sodium Dodecyl Sulphate-Polyacrylamide Gel Electrophoresis (SDS-PAGE). Subsequently, the samples were digested using trypsin enzyme from Promega (Madison, WI, USA) and labeled with different iTRAQ tags.

The labeled sample fragments were separated through the Shimadzu LC-20AB liquid chromatography (LC) system before being subjected to liquid separation in the Thermo Scientific™ UltiMate™ 3000 UHPLC system. Finally, the samples were analyzed in the Thermo Fisher Scientific Q-Exactive HF X hybrid quadrupole-Orbitrap mass spectrometer. The resulting mass spectrometry raw data were converted and identified with the UniProt plant database (https://www.uniprot.org/) via the Mascot software (v2.3.02).

Quantitative analysis of isobaric tag-labelled peptides was conducted using the automated software IQuant [39]. This software incorporates Mascot Percolator, an efficacious machine-learning method that rescores database search outcomes to provide reliable significance measures. To evaluate peptide confidence, peptide-spectrum matches (PSMs) were initially filtered at a 1% false discovery rate at the PSM level. The identified peptide sequences were then assembled into a confident set of proteins, following the parsimony principle. To manage the false-positive rate at the protein level, a protein FDR of 1%, based on the picked protein FDR strategy [40], was also calculated subsequent to protein inference (protein-level FDR ≤ 0.01). The process of protein quantification encompassed several stages, including protein identification, tag impurity correction, data normalization, missing value imputation, protein ratio calculation, statistical analysis, and results presentation. Comprehensive functional annotation of the identified proteins was performed using Gene Ontology (GO), Eukaryotic Orthologous Groups (KOG), and Kyoto Encyclopedia of Genes and Genomes (KEGG) pathways.

Proteins with a 1.2-fold change and *Q-value* less than 0.05 were determined as differentially expressed proteins in a single replicate. A comparison was subsequently made between these differentially expressed proteins and differentially expressed genes using Venn diagrams. Further, GO and KEGG enrichment analyses were performed on differentially expressed proteins (DEPs) with a threshold FDR value of less than 0.05.

### Examination of positive selection within hypoxia-responsive related genes

In this study, a positive selection analysis was conducted using 10 coding sequences (CDS) derived from both wild and cultivated tomato species. Additional genomic data, comprising eight diverse flowering plant species, including *Actinidia chinensis*, *Amborella trichopoda*, *Arabidopsis thaliana*, *Ipomoea nil*, *Medicago truncatula*, *Oryza sativa*, *Vitis vinifera* and *Zostera marina*, were retrieved from Phytozome (https://phytozome-next.jgi.doe.gov). The preliminary stage of the analysis required the exclusion of isoforms and the translation of the CDS into corresponding protein sequences. Subsequently, these protein sequences were clustered into orthologue groups (OGs) using the OrthoFinder (v2.5.4) [41]. The protein sequences obtained from each OGs were aligned into multiple sequence alignment files by MAFFT (v7.508) [42]. PAL2NAL was used to convert the multiple sequence alignment of proteins to the corresponding DNA sequences [43].

Phylogenetic trees of the multi-copy gene families were constructed with IQTREE (v2.2.0.3) [44]. These gene trees were rooted based on the phylogeny and consequently decomposed into single-copy gene trees via PhyloTracer (https://github.com/YiyongZhao/PhyloTracer), retaining only those with species coverage exceeding 50%. Furthermore, only those single-copy gene trees encompassing hypoxia-responsive related genes from both wild and cultivated tomatoes (either differentially expressed genes or proteins from transcriptomic and proteomic analyses in this study) were used for gene-positive selection analyses. The selected single-copy gene trees and their corresponding aligned CDS sequences were subjected to the ete3-evol tool to identify genes under positive selection, by utilizing the b_free b_neut M0 models [45].

### Protein-protein interaction (PPI) network analysis for differentially expressed genes (DEGs) and differentially expressed proteins (DEPs)

We performed PPI network analysis to investigate the interactions among differentially expressed genes (DEGs) and differentially expressed proteins (DEPs) in the tomato cultivars FZZ and T178. First, we downloaded the *Solanum lycopersicum* PPI dataset (https://stringdb-downloads.org/download/protein.physical.links.full.v12.0/4081.protein.physical.links.full.v12.0.txt.gz) from the STRING database (version 12.0) [46]. The protein sequences corresponding to the DEGs and DEPs were inputted into the STRING platform using the “Protein by Sequence” module to retrieve the associated gene IDs. Using these gene IDs, we extracted the PPI networks for each gene from the *Solanum lycopersicum* PPI database, including the corresponding nodes and combined scores (only PPI networks with a score ≥ 700 were selected). The PPI networks were visualized using Cytoscape (version 3.10.3) [47]. We merged the networks using the built-in “Merge” module and calculated network properties such as degree, betweenness centrality and clustering coefficient using the “Network Analyzer” module. Functional enrichment analysis of the network was performed using the ClueGO plugin (version 2.5.10) [48] in Cytoscape. To identify key hub genes, we employed the cytoHubba plugin (version 0.1) [49] in Cytoscape, selecting the top 20 hub genes in the merged network for comparative analysis.

## Results

### Quantitative evaluation of tomato seedlings under aerated/hypoxic treatments

To assess the hypoxia sensitivity of two tomato varieties, we established two treatment groups: a normoxic group (nutrient solution enriched with air; T178-A and FZZ-A, respectively) and a hypoxic group (nutrient solution enriched with nitrogen; T178-H and FZZ-H, respectively), and cultivated seedlings for 12 days. At harvest, 12 seedlings per group were randomly selected for trait measurements, including leaf number, plant height, stem diameter, chlorophyll content, root length, stem dry weight, root dry weight, and total dry weight (Supplementary Table S1).

Under hypoxic conditions, root length was the only trait significantly affected in both FZZ and T178. Specifically, the average root length of FZZ decreased from 24.55 ± 1.41 cm under normoxia to 15.76 ± 0.51 cm under hypoxia. Similarly, T178 showed a reduction from 25.94 ± 1.00 cm under normoxia to 15.27 ± 0.64 cm under hypoxia. Thus, hypoxia reduced root length by 35.80% in FZZ and 41.13% in T178 (Fig. 1B). These results confirm that hypoxia markedly inhibits root growth in both varieties. To further quantify the sensitivity differences between FZZ and T178, we calculated membership function values [50] for various growth traits under both normoxic and hypoxic conditions by using the formula: $\:R\left({X}_{i}\right)=\frac{{X}_{i}-{X}_{\text{m}\text{i}\text{n}\:}}{{X}_{\text{m}ax\:}-{X}_{\text{m}in\:}}$, and then compared the average total membership function values (Supplementary Table S2). Under normoxic conditions, FZZ’s average membership function value exceeded 0.7, indicating a high sensitivity to normal aeration, whereas T178’s value remained below 0.3, suggesting low sensitivity to the same conditions. Under hypoxia, FZZ’s average membership function value ranged from 0.6 to 0.7, indicating relatively high hypoxia sensitivity, while T178’s value remained below 0.3, signifying low hypoxia sensitivity. Thus, wild tomato T178 is inherently less sensitive to oxygen availability than cultivated tomato FZZ, and its growth is less impacted by hypoxia.

In summary, although both FZZ and T178 experienced significant root growth inhibition under hypoxia, with T178 showing a slightly greater proportional reduction, membership function [50] analysis revealed that FZZ is more sensitive to changes in oxygen availability in general, whereas T178 demonstrates robust tolerance to low-oxygen conditions. These findings underscore the distinct physiological responses of wild and cultivated tomatoes to hypoxia and highlight the need for further investigation of the molecular mechanisms underlying hypoxia tolerance. Ultimately, such insights may inform breeding strategies aimed at improving the stress resilience of cultivated tomato varieties.

### Identification of differentially expressed genes (DEGs) in wild and cultivated tomato from transcriptomic data

To investigate the molecular effects of hypoxia on tomato roots, RNA-seq technology was utilized to investigate the root transcriptomes of both wild type of tomato T178 and cultivated tomato FZZ under normoxic and hypoxic conditions. Two independent biological replicates were performed for each sample. The RNA-seq outcomes for the eight samples are summarized in Supplementary Table S3. Each sample yielded between 46.38 and 49.19 million raw sequence reads. Upon adapter removal and low-quality base filtering, each biological replicate obtained between 42.11 and 44.52 million high-quality reads. Bases with a quality score ≥ 20 and ≥ 30 accounted for more than 95% and 85% of the total bases, respectively. For T178, 86.98-87.28% of the total reads were mapped onto the wild tomato reference genome (accessible at https://www.omicshare.com/tools). For cultivated tomato FZZ, 94.1-94.45% of total reads were mapped onto the cultivated tomato reference genome *S. lycopersicum* ITAG-v4.0 which was downloaded from the plant comparative genomics portal Phytozome v13 (https://data.jgi.doe.gov/refine-download/phytozome?organism=Slycopersicum). Transcriptome coverage statistics were performed, revealing a peak coverage between 90 and 100% accounting for more than 32-38% (Supplementary Fig. S1A), indicating high sequencing quality and reliable data source suitable for downstream analysis. Correlation coefficients exceeding 0.97 (Supplementary Fig. S1B) were submitted to statistical analyses of the two biological replicates per sample, further attesting to the data reliability.

**Fig. 2:**
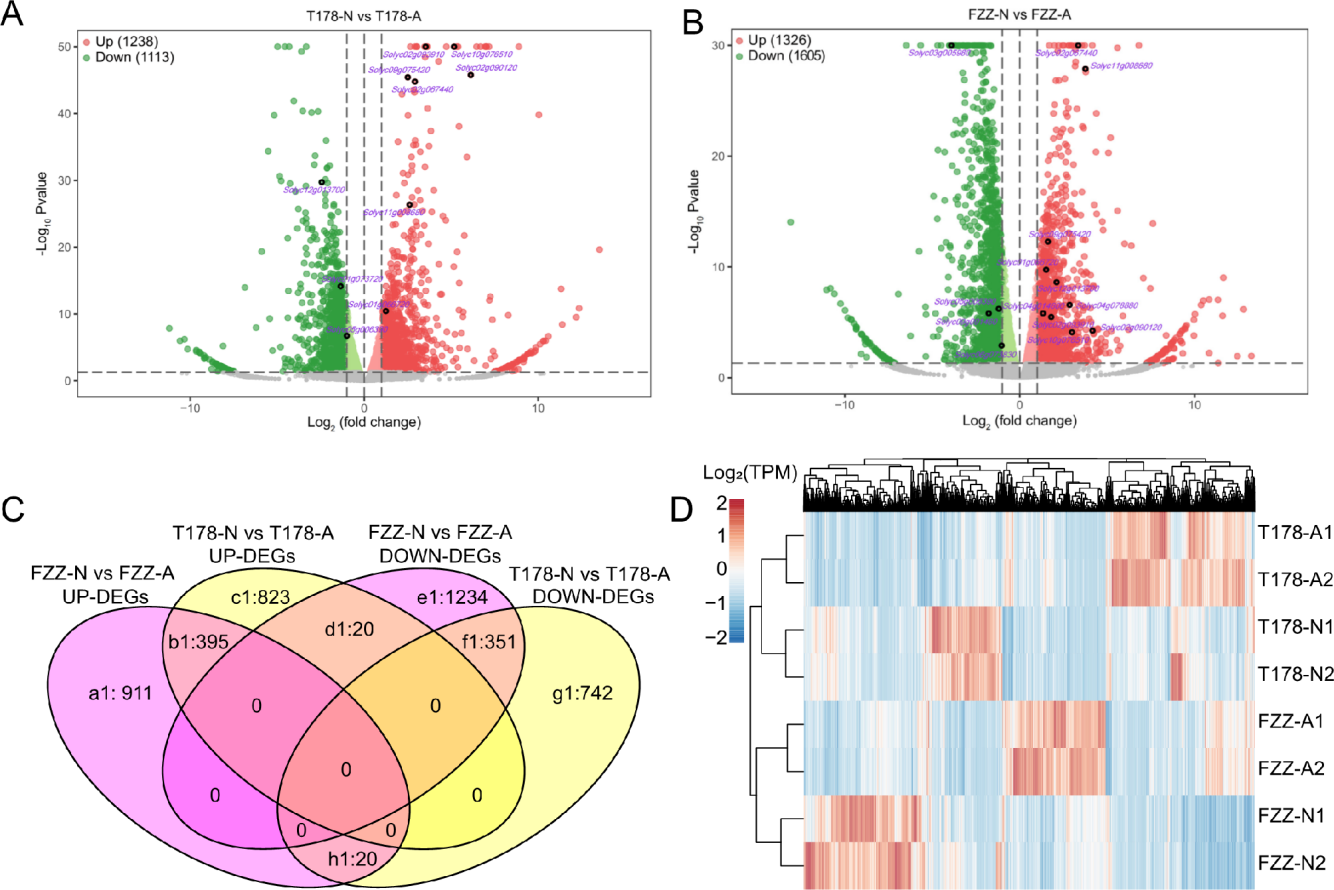
Volcano plot of differentially expressed genes. (**A**) Volcano plot comparing wild tomato under hypoxia (T178-N) versus air (T178-A). (**B**) Volcano plot comparing cultivated tomato under hypoxia (FZZ-N) versus air (FZZ-A). Genes up-regulated (red) exhibit a fold change > 2 and P-value < 0.05, and down-regulated genes (green) display a fold change < 0.5 and P-value < 0.05. Purple represents homologous genes in tomatoes related to Arabidopsis’ hypoxia-responsive genes. Each plot depicts a volcano plot of log2 fold-change (x-axis) versus -log_10_*Q-value* (y-axis, representing adjusted statistical significance). *Q-value* < 0.05 is set as the significant threshold for differentially expression. The red and green dots indicate points of interest that display both large-magnitude fold changes as well as high statistical significance. Red dots indicate significantly up-regulated proteins which passed the screening threshold. Dots in green mean significant down-regulated proteins which passed the screening threshold. And gray dots are non-significant differentially expressed proteins. (**C**) Venn diagram showing up-regulated and down-regulated genes in wild and cultivated tomatoes under hypoxia compared to air. (**D**) Heatmap depicting the clustering of differentially expressed genes. The Y-axis represents two biological replicates of each sample, whereas the X-axis represents significantly expressed differentially expressed genes. The heatmap is colored based on the log₂(TPM + 1) of each gene across samples

We compared transcriptome data from two groups, T178-N vs. T178-A and FZZ-N vs. FZZ-A, to identify differentially expressed genes (DEGs). To facilitate the subsequent enrichment analysis, we completed the ID match between wild tomato DEGs and cultivated tomato DEGs using the Blast program for two-way optimum comparison. A total of 2351 DEGs were found between T178-N vs. T178-A, including 1238 up-regulated genes and 1113 down-regulated genes (Fig. 2A and Supplementary Table S4). Comparison between FZZ-N and FZZ-A revealed 2931 DEGs, of which 1326 were up-regulated and 1605 were down-regulated (Fig. 2B and Supplementary Table S5). A literature search indicated that among the FZZ group, 15 DEGs were known oxygen-related genes, whereas only seven DEGs were known oxygen-related genes in T178 (Supplementary Table S6). Furthermore, the Venn diagram (Fig. 2C) illustrated that 911 DEGs were uniquely up-regulated in FZZ-N compared to FZZ-A (Fig. 2C a1); 823 DEGs were uniquely up-regulated in T178-N compared to T178-A (Fig. 2C c1); 1234 DEGs were uniquely down-regulated in FZZ-N relative to FZZ-A (Fig. 2C e1); and 742 DEGs were uniquely down-regulated in T178-N relative to T178-A (Fig. 2C g1). Additionally, 395 DEGs were concurrently up-regulated in both T178-N vs. T178-A and FZZ-N vs. FZZ-A overlap genes (Fig. 2C b1), and 351 DEGs were concurrently down-regulated in both comparisons (Fig. 2C f1). Overall, the results showed a strong correlation between replicate samples and the high reliability of the data. The expression patterns showed significant differences under different oxygen conditions (Fig. 2D).

In conclusion, this study successfully identified DEGs in the roots of wild and cultivated tomatoes under hypoxic conditions using RNA-seq technology. The high-quality transcriptomic data provide a solid foundation for understanding the molecular responses of different tomato varieties to hypoxia. These findings are expected to contribute to revealing the underlying mechanisms of plant growth regulation under hypoxia and provide important insights for future strategies aimed at improving stress resistance in various tomato cultivars.

### Differentially expressed genes (DEGs) and enrichment analysis of GO and KEGG in wild and cultivated tomatoes

**Fig. 3:**
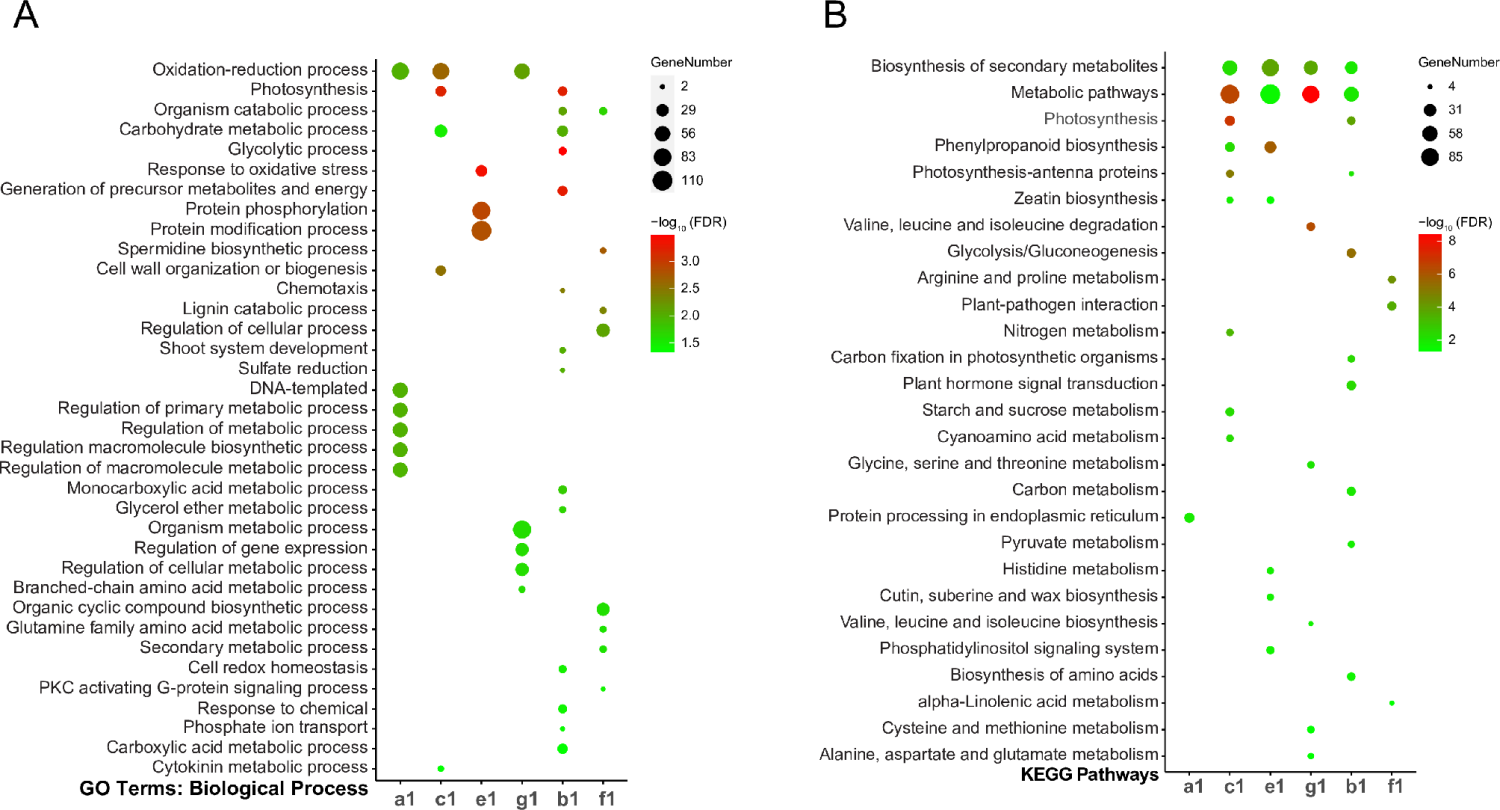
Differential Gene GO, KEGG Enrichment. (**A**) Bubble chart of GO enrichment analysis (FDR < 0.05), outlining the biological processes of differentially expressed genes in wild and cultivated tomatoes under various conditions. (**B**) Bubble chart of KEGG pathway enrichment analysis (FDR < 0.05), detailing the metabolic pathways of differentially expressed genes in wild and cultivated tomatoes under diverse conditions

To elucidate the molecular mechanism of wild and cultivated tomatoes under hypoxia, a comprehensive exploration of differentially expressed genes (DEGs) was conducted using GO and KEGG analysis across the specified groups (Fig. 2C, a1, b1, c1, e1, f1, g1). A total of 270 GO terms in six groups were assigned to 198 unique GO categories, encompassing 101 unique biological processes, unique 20 cellular components, and unique 77 molecular functions (Supplementary Table S7). Specifically, the up-regulated DEGs, those of FZZ-N vs. FZZ-A were mainly associated with redox processes (GO:0055114) and DNA-templated transcription (GO:0006351) (Fig. 3A a1), whereas those of T178-N vs. T178-A were mainly abundant in redox processes (GO:0055114) and carbohydrate metabolism (GO:0005975) (Fig. 3A c1). Among the down-regulated DEGs, FZZ was mainly found in protein modification processes (GO:0036211) and protein phosphorylation (GO:0006468) (Fig. 3A e1). In T178, on the other hand, it was only enriched in single-organism metabolic processes (GO:0044710) and redox processes (GO:0055114) (Fig. 3A g1). Moreover, in T178-N vs. T178-A and FZZ-N vs. FZZ-A overlap, up-regulated DEGs were most abundant in carbohydrate metabolism processes (GO:0005975) and carboxylic acid metabolism processes (GO:0019752) (Fig. 3A b1), whereas down-regulated DEGs were mainly enriched in cellular process regulation (GO:0050794) and organic ring compound biosynthetic processes (GO:1901362) (Fig. 3A f1).

KEGG enrichment analysis revealed that the DEGs specifically up-regulated in the comparison of FZZ-N vs. FZZ-A were significantly enriched in the pathway of endoplasmic protein processing in the endoplasmic reticulum (ko04141) (Supplementary Table S8; Fig. 3B a1). The down-regulated DEGs in FZZ-N vs. FZZ-A (Fig. 3B e1), T178-N vs. T178-A (Fig. 3B c1) and T178-N vs. T178-A (Fig. 3B g1), as well as the up-regulated ones in T178-N vs. T178-A with FZZ-N vs. FZZ-A (Fig. 3B b1) were enriched mainly in biosynthesis of secondary metabolites (ko01110) and metabolic pathways (ko01100). However, for the down-regulated DEGs in both T178-N vs. T178-A and FZZ-N vs. FZZ-A (Fig. 3B f1), they were mainly enriched in arginine and proline metabolism (ko00330), plant-pathogen interaction (ko04626), and alpha-linolenic acid metabolism (ko00592).

In conclusion, the DEGs identified through GO and KEGG enrichment analyses provide valuable insights into the adaptive responses of wild and cultivated tomatoes to hypoxia. The significant enrichment of genes involved in redox processes and carbohydrate metabolism highlights the crucial metabolic adjustments these plants undergo to cope with low oxygen levels. Understanding these molecular mechanisms is essential for developing strategies to enhance hypoxia tolerance in tomato cultivars, thereby improving their growth and productivity in challenging environments.

### Identification of differentially expressed proteins (DEPs) in wild and cultivated tomatoes from proteomic data

In the present study, we utilized the Isobaric Tags for Relative and Absolute Quantitation (iTRAQ) technology to investigate the proteomic landscape of our chosen sample set. This set consisted of four pooled samples, incorporating both wild and cultivated tomato variants exposed to both normoxic and hypoxic environmental conditions. This, in turn, formed a total of four distinct experimental groups. For each of these groups, we executed duplicate biological replicate experiments to ensure the reliability and reproducibility of our findings. Proteins that were recognized within any of these replicates, exhibiting a false discovery rate (FDR) below the threshold of 0.01, were operationally defined as “proteins” in the context of this study.

**Fig. 4:**
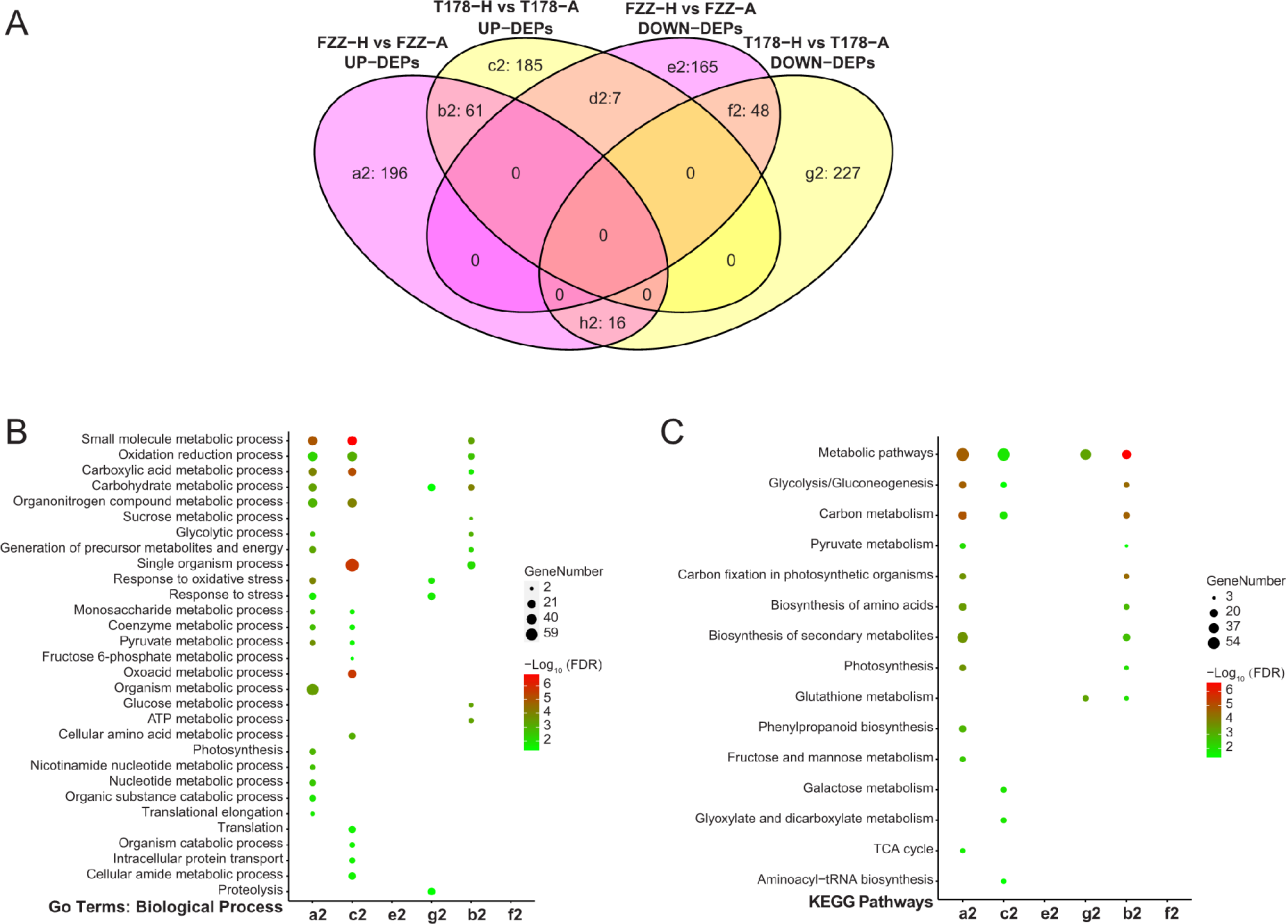
Differential protein analysis. (**A**) Venn diagram showing the up-regulated and down-regulated proteins in wild and cultivated tomatoes under hypoxia compared to air. (**B**&**C**) Bubble charts of GO and KEGG enrichment analyses (FDR < 0.05) illustrate the biological processes and metabolic pathways of differentially expressed proteins in wild and cultivated tomatoes under various conditions

By comparing the proteomic data of T178-A, T178-N, FZZ-A, and FZZ-N, there were 544 differentially expressed proteins (DEPs), including 291 up-regulated proteins and 253 down-regulated proteins in T178-N vs. T178-A (Supplementary Table S9). FZZ-N vs. FZZ-A showed 493 DEPs, with 220 up-regulated and 273 down-regulated (Fig. 4A and Supplementary Table S10). We found some correlations in the expression pattern of DEPs between different samples (Fig. 4B). For example, in the FZZ-N vs. FZZ-A group, there were 196 DEPs specifically up-regulated (Fig. 4B a2) and 165 specifically down-regulated DEPs (Fig. 4B e2), whereas there were 185 DEPs specifically up-regulated (Fig. 4B c2) and 227 specifically down-regulated DEPs (Fig. 4B g2) in the T178-N vs. T178-A. The data also showed that 61 of the FZZ vs. T178 overlapping portion of the protein set were up-regulated DEPs (Fig. 4B b2) and 48 DEPs were generally down-regulated (Fig. 4B f2), respectively.

In conclusion, the identification of DEPs in both wild and cultivated tomatoes reveals significant proteomic changes in response to hypoxia. The up regulation of certain proteins may indicate adaptive mechanisms employed by the plants to mitigate the adverse effects of low oxygen levels. This proteomic analysis complements our transcriptomic findings, highlighting the coordinated response at both mRNA and protein levels, which is crucial for understanding how these tomato variants cope with hypoxic conditions. These insights can inform breeding strategies aimed at enhancing hypoxia tolerance in tomato crops, ultimately contributing to improved agricultural productivity in environments where oxygen availability is limited.

### GO and KEGG enrichment analysis of differential expression protein (DEPs) from wild vs. cultivated tomatoes

We enriched the genes encoding differentially expressed proteins (DEPs) and all DEPs were categorized into 245 GO terms, including 129 biological processes, 28 cellular components, and 88 molecular functions (Supplementary Table S11). Among them, DEPs specifically up-regulated in FZZ-A vs. FZZ-N (Fig. 4C a2) and T178-N vs. T178-A (Fig. 4C c2), as well as DEPs up-regulated in both T178 vs. FZZ (Fig. 4C b2), were mainly involved in single-organism metabolic processes (GO:0044710) and redox processes (GO:0055114). The down-regulated DEPs in T178-N vs. T178-A were mainly involved in carbohydrate metabolic processes (GO:0005975) and stress responses (GO:0006950) (Fig. 4C g2). It is noteworthy that no enriched categories were found in the DEPs down-regulated of FZZ-N vs. FZZ-A (Fig. 4C e2) and the DEPs down-regulated in both T178 vs. FZZ (Fig. 4C f2), and this part of the genes may not have been validated for the relevant functions.

KEGG analysis revealed a total of 28 pathways (Supplementary Table S12). The DEPs specifically up-regulated in FZZ-N vs. FZZ-A (Fig. 4D a2) and those up-regulated in T178-N vs. FZZ-N (Fig. 4D b2) were mainly concentrated in the biosynthesis of secondary metabolites (ko01110) and metabolic pathways (ko01100). The DEPs specifically up-regulated in T178-A vs. DEPs were mainly associated with metabolic pathways (ko01100) and carbon metabolism (ko01200) (Fig. 4D c2). Specific down-regulated DEPs in T178-N vs. T178-A were mainly enriched in metabolic pathways (ko01100) and glutathione metabolism (ko00480) (Fig. 4D g2). No enrichment terms were found for DEPs specifically down-regulated in the FZZ-N vs. FZZ-A (Fig. 4D e2) and for DEPs exhibiting down-regulation in both T178 and FZZ (Fig. 4D f2).

In conclusion, the GO and KEGG enrichment analyses of the differentially expressed proteins provide significant insights into the molecular mechanisms underlying the hypoxic response in wild and cultivated tomatoes. The up-regulation DEPs related to metabolic processes and redox functions suggests a robust adaptive response to low oxygen levels, highlighting potential pathways that could be targeted for enhancing hypoxia tolerance in tomato crops. Conversely, the down-regulated proteins in response to hypoxia indicate compromised metabolic functions, which may negatively affect plant growth and resilience. Understanding these protein-level changes can guide future research aimed at developing hypoxia-resistant tomato varieties, ultimately supporting sustainable agricultural practices in oxygen-limited environments.

### Integrated analysis of transcriptomic and proteomic profiles in wild and cultivated tomatoes

**Fig. 5:**
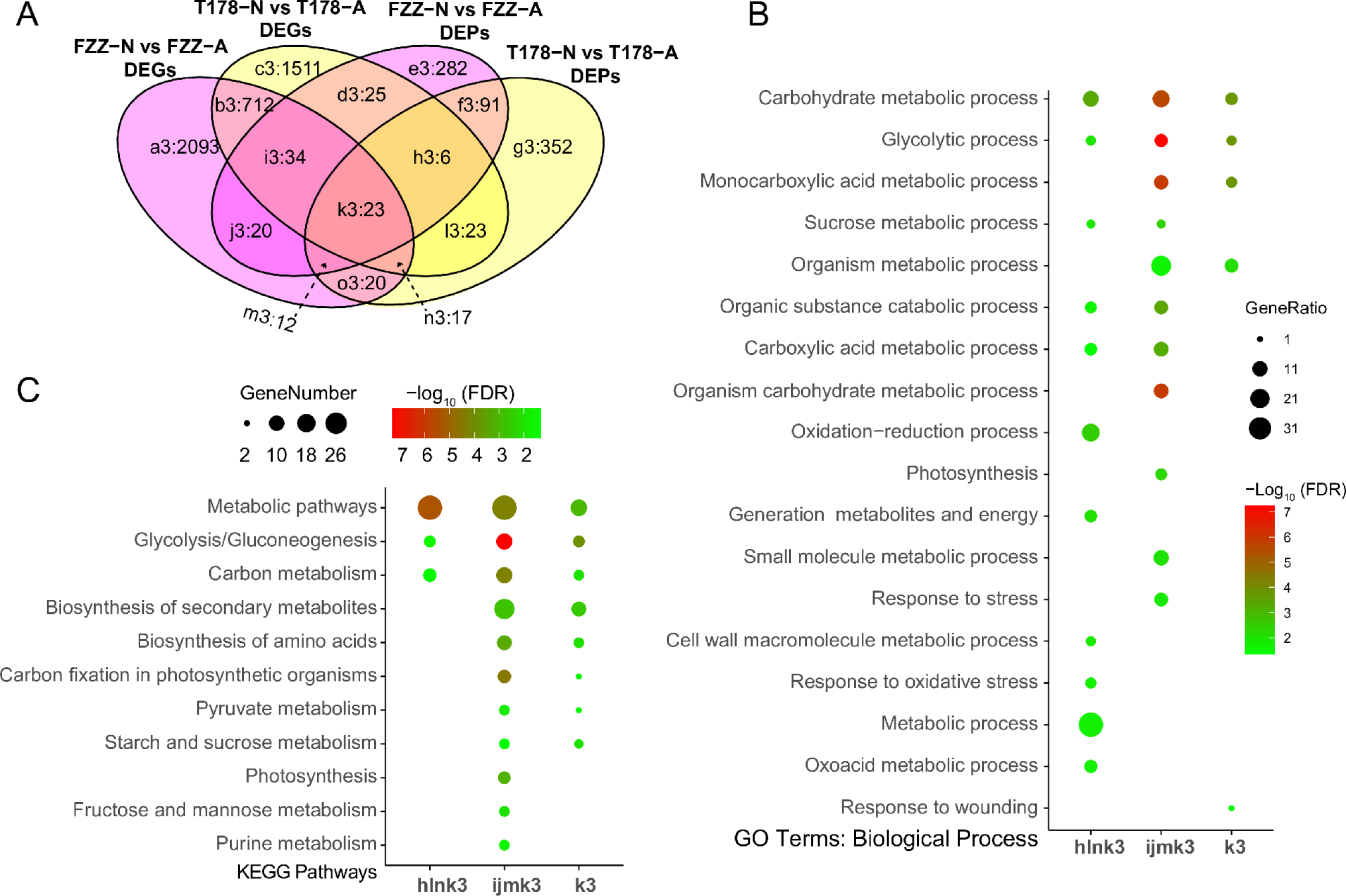
Combined analysis of differential genes and differential proteins. (**A**) Venn diagram showing the overlap between differentially expressed genes and proteins in wild and cultivated tomatoes under hypoxia compared to air. (**B**&**D**) Bubble charts of GO and KEGG enrichment analyses (FDR < 0.05) illustrate the biological processes and metabolic pathways of differentially expressed genes and proteins in wild and cultivated tomatoes under various conditions

To further validate the relationship between the tomato transcriptome and proteome, we performed a conjoint analysis of differentially expressed genes (DEGs) and differentially expressed proteins (DEPs). A total of 89 overlapping genes were found in FZZ-N vs. FZZ-A (Fig. 5A ijmk3), and 69 overlapping genes were also found in T178-N vs. T178-A (Fig. 5A hlnk3). 23 genes were identified that were all present in four subgroups including DEGs and DEPs in T178 and FZZ (Fig. 5A k3).

GO and KEGG enrichment analyses of the above groups of genes were performed separately. GO enrichment showed that they were distributed across 154 GO terms, including 90 biological processes, 13 cellular components, and 51 molecular functions (Supplementary Table S13). The factors in the FZZ-N vs. FZZ-A group (Fig. 5B ijmk3) and those consistently found in two Omics analysis comparisons of T178 and FZZ (Fig. 5B k3) were mainly enriched in single-organism metabolic processes (GO:0044710) and carbohydrate metabolic processes (GO:0005975). In contrast, for the T178-N vs. T178-A group, the factors found in both global omics were most enriched in metabolic processes (GO:0008152) and redox processes (GO:0055114) (Fig. 5B hlnk3). Kyoto Encyclopedia of Genes and Genomes (KEGG) pathway enrichment analysis demonstrated common pathway enrichment between the transcriptome and proteome in both FZZ-N vs. FZZ-A and T178-N vs. T178-A comparisons (Fig. 5C and Supplementary Table S14). These enriched pathways included Glycolysis/Gluconeogenesis (ko00010), Metabolic pathways (ko01100), Biosynthesis of secondary metabolites (ko01110), Biosynthesis of amino acids (ko01230), Starch and sucrose metabolism (ko00500), Carbon metabolism (ko01200), Carbon fixation in photosynthetic organisms (ko00710), and Pyruvate metabolism (ko00620). Notably, the specific factors identified in both the transcriptome and proteome of FZZ-N vs. FZZ-A showed additional enrichment in Photosynthesis (ko00195), Fructose and mannose metabolism (ko00051), and Purine metabolism (ko00230) (Fig. 5C).

In conclusion, the integrated analysis of transcriptomic and proteomic data highlights a significant overlap between the two omics layers in both wild and cultivated tomatoes, particularly under hypoxic conditions. The enrichment of genes and pathways related to metabolic processes and carbohydrate metabolism underscores the adaptive strategies these plants employ to cope with low oxygen environments. Furthermore, the identification of specific pathways shared between DEGs and DEPs provides a valuable framework for future research aimed at enhancing hypoxia tolerance in tomato crops. Understanding these molecular interactions could lead to the development of improved varieties that better withstand environmental stressors, ultimately contributing to agricultural sustainability and food security.

### Positive selection analysis of differentially expressed genes and proteins in wild and cultivated tomatoes

**Fig. 6:**
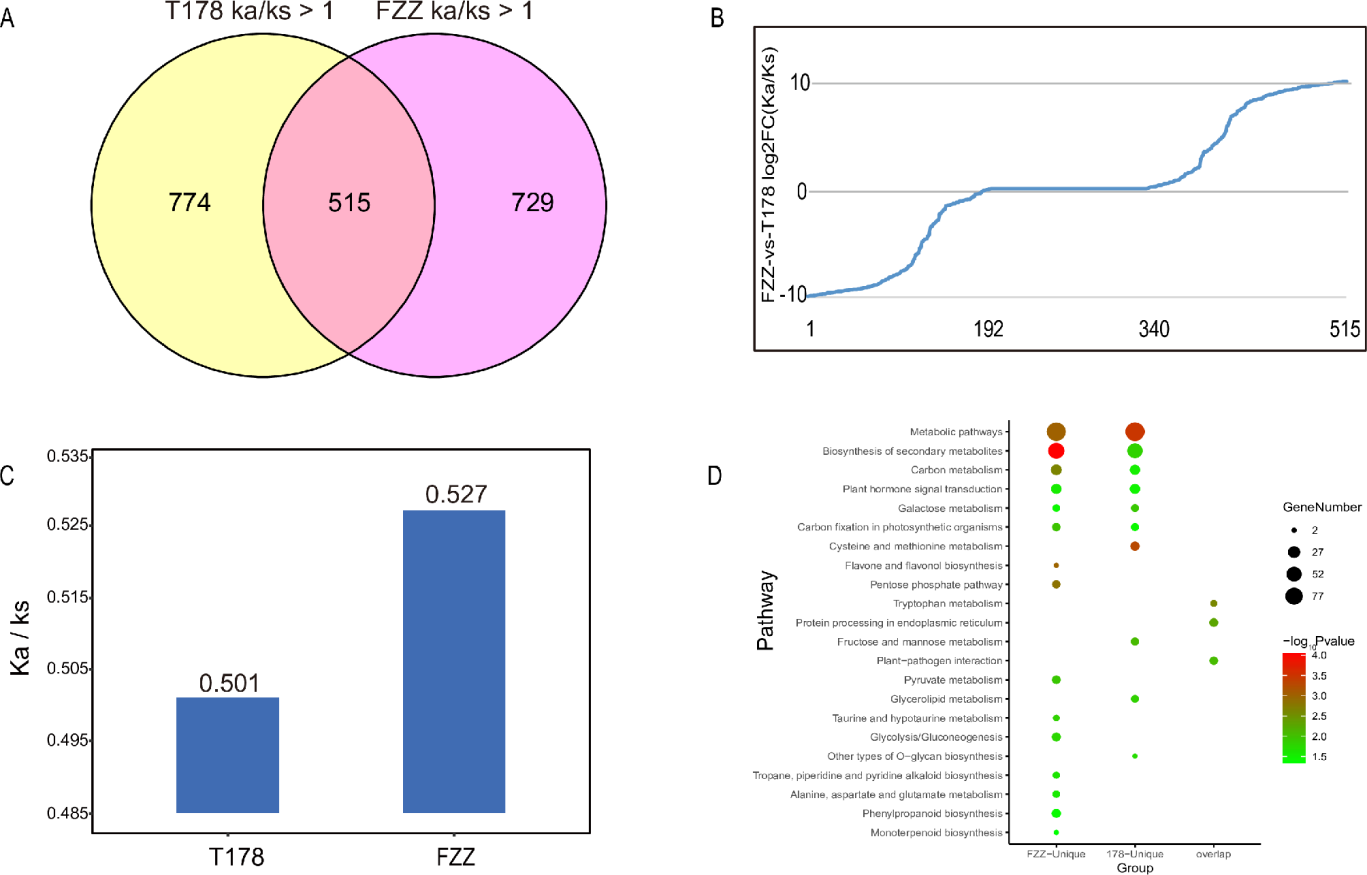
Summary of the positive selection analysis for DEGs and DEPs. (**A**) Venn diagram showing the overlap of genes and proteins under positive selection in wild and cultivated tomatoes. (**B**) The ratio of genes under positive selection in both wild and cultivated tomatoes. (**C**) Ka/Ks values of differentially expressed genes and proteins in wild and cultivated tomatoes. (**D**) Summary of KEGG enrichment analysis for genes under positive selection

Natural selection and anthropogenic domestication may lead to adaptive evolution in plants in which specific traits are retained and exhibit varying degrees of selection traces at the gene level. To further explore the relationship between environment and hypoxia stress adaptation traits during tomato evolution. We analyzed the selection pressure of genes found in the transcriptome and proteome with homologous gene clusters as the smallest unit. The results showed that 1289 genes were positively selected in the T178 group and 1244 genes in the FZZ group (Fig. 6A, Supplementary Table S15). In the T178 group, 774 specific genes were positively selected, while 729 were positively selected in the FZZ group. A total of 515 genes were positively selected in both groups. To further understand the potential differences between T178 and FZZ during selection, we compared these 515 genes that were all subject to positive selection pressure; 192 and 175 genes in FZZ and T178, respectively, showed stronger positive selection pressure, and the other 148 genes showed equal strength of positive selection pressure in both species (Fig. 6B). Overall, we observed a higher degree of positive selection in FZZ than in T178 (Fig. 6C). We also found only a small overlap of positively selected genes with DEG and DEP (Supplementary Fig. S2). In addition, GO and KEGG analysis showed that the unique positively selected genes in FZZ and T178 were mainly distributed in carbon metabolism hormone synthesis, and energy metabolism-related pathways, whereas the overlapping genes in FZZ and T178 were distributed in three pathways, namely, Tryptophan metabolism, Protein processing in endoplasmic reticulum, and Plant-pathogen interaction (Fig. 6D and Supplementary Fig. S3).

In summary, this positive selection analysis highlights the adaptive evolution of wild and cultivated tomatoes in response to environmental stresses, particularly hypoxia. Although a higher degree of positive selection was observed in FZZ, the known hypoxia tolerance of T178 suggests that other mechanisms, such as gene expression regulation or protein modifications, may play a more critical role in its adaptation to low oxygen conditions. These differences may reflect distinct evolutionary strategies, with FZZ relying more on genetic variation and T178 utilizing additional physiological or molecular adaptations to cope with hypoxia. Additionally, the pathways enriched with positively selected genes provide insight into the molecular mechanisms underlying the plants’ adaptive traits. Understanding these genetic and proteomic adaptations can inform breeding strategies aimed at enhancing hypoxia tolerance in cultivated tomatoes, ultimately contributing to sustainable agricultural practices in challenging environments.

### Transcriptional differences in pathways of plant hormone signal transduction, photosynthesis, energy metabolism, and plant-pathogen interactions between wild and cultivated tomato under hypoxic and ambient air conditions

In this study, most of the differentially expressed genes (DEGs) were enriched in multiple metabolic pathways, particularly those related to phytohormones, photosynthesis, energy metabolism, and pathogen interactions, which are closely related to plant growth and development, drew our attention.

**Fig. 7:**
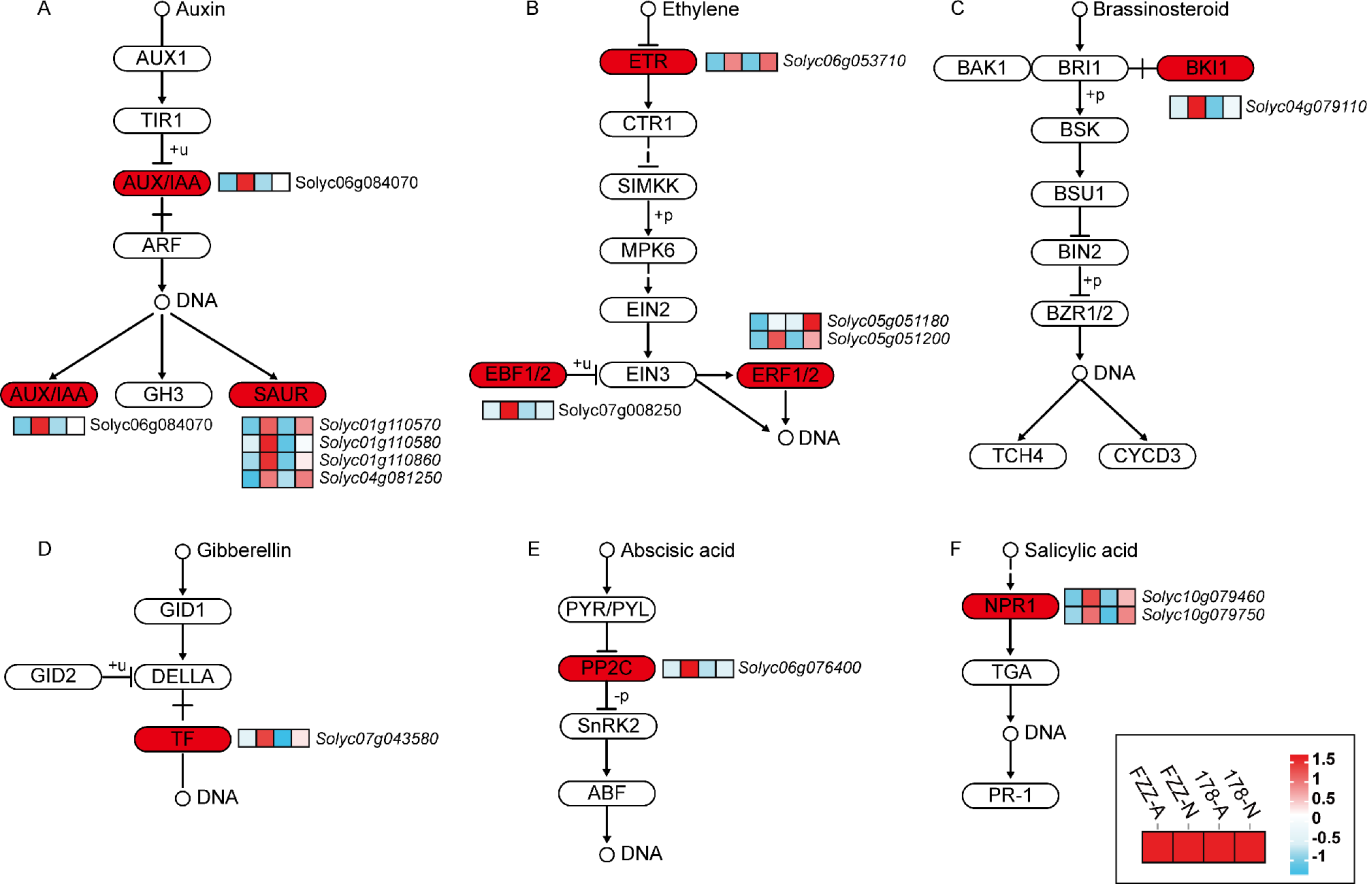
Hormonal pathways associated with the up-regulated expression of hypoxia stress genes. Hormonal regulation pathway analysis of up-regulated genes common to wild and cultivated tomatoes under hypoxia compared to air

Initially, we analyzed the relevant genes of Phytohormone Signaling Pathways in the enrichment analysis for T178 and FZZ (Fig. 7 and Supplementary Table S16). In the auxin pathway (Fig. 7A), the auxin/indole-3-acetic acid (*AUX/IAA*) gene (*Solyc06g084070*) was significantly more up-regulated in FZZ than in T178; among the four small auxin-up RNA (*SAUR*) genes, *Solyc01g110580* and *Solyc01g110860* were significantly more up-regulated in FZZ than in T178, and the expression levels of the other two genes were similar. In the ethylene synthesis pathway (Fig. 7B), the expression levels of the *ETR* gene (*Solyc06g053710*) were comparable in FZZ and T178, whereas the *EBF1/2* gene (*Solyc07g008250*) and the two genes encoding *ERF1/2* (*Solyc05g051180* and *Solyc05g051200*) were both up regulated. The expression of the chlorophyll synthesizing gene *BKI1* (Fig. 7C), the gibberellin synthesizing gene TF (Fig. 7D), and the gibberellin synthesizing gene *PP2C* (Fig. 7E) was higher in FZZ-N. The expression of the salicylic acid synthesis gene *NPR1* (Fig. 7F) was increased in both FZZ-N and T178-N. Overall, there was a trend of up-regulation of the expression for the relevant genes in the above hormonal pathways after hypoxia treatment.

Secondly, we found significant changes in genes related to the photosynthesis pathway for FZZ and T178 under hypoxic conditions (Supplementary Fig. S4, Supplementary Fig. S6 and Supplementary Table S17) [51]. After hypoxia treatment, genes encoding photosystem II (PSII) proteins (*psbO*, *psbP*, *psbQ*, *psbS*, *psbW*, and *psb27*), genes encoding photosystem I (PSI) proteins (*psaD*, *psaE*, *psaG*, *psaK*, *psaL*, *psaN*, *psaO*, and *psaH*), and genes encoding PCs as well as the *ATPF0B* and *ATPF1G* genes were significantly higher expressed in T178-N than FZZ. Among the genes encoding Fd proteins, the two *petF* genes, *Solyc11g068430* and *Solyc10g075160*, were significantly up regulated in T178, except for *Solyc11g006910*.

Thirdly, in terms of energy metabolism, we focused on two pathways, carbon metabolism, and glycolysis, which were enriched with more genes. Our results showed that, in addition to the *GAPDH* gene, hypoxia treatment significantly induced the expression of other related genes in glycolysis and carbon metabolism pathways in T178 and FZZ (Supplementary Table S18). The expression of the *GAPDH* (*Solyc12g094640*) gene was significantly increased in the hypoxia-tolerant cultivar T178, but the expression of the *GAPDH* gene was down-regulated under hypoxic conditions in the cultivar FZZ. Overall, genes involved in the glycolytic pathway exhibited more pronounced differential expression in T178, particularly *Solyc02g077240* (T178: fold change [FC] = 39.88; FZZ: FC = 5.71) and *Solyc10g076510* (T178: FC = 28.09; FZZ: FC = 7.92). In contrast, carbon metabolism pathways were upregulated in both species, though they showed minimal fold changes in differential expression.

Finally, at the intersection of FZZ and T178 down-regulated genes, we found that “plant-pathogen interaction (ko04626)” was the most highly enriched pathway, with the most enriched genes (Fig. 3b f1). Hypoxia treatment resulted in a significant down-regulation of the expression of genes related to the phytopathogen pathway in the cultivated tomato variety FZZ and the wild tomato variety T178. Notably, the wild variety exhibited higher gene expression levels compared with FZZ (Supplementary Table S19).

Overall, these transcriptional differences underscore the adaptive mechanisms employed by wild and cultivated tomatoes in response to hypoxic conditions. The up regulation of hormone signaling pathways, photosynthetic genes, and energy metabolism in T178 suggests a robust physiological response to hypoxia, potentially enhancing survival and resilience. Conversely, the down regulation of plant-pathogen interaction genes may reflect a trade-off in resource allocation under stress conditions, highlighting the complexities of plant responses to environmental challenges. Understanding these pathways can provide valuable insights for breeding programs aimed at improving hypoxia tolerance and disease resistance in cultivated tomatoes.

### Protein-protein interaction network analyses for differentially expressed genes (DEGs) and differentially expressed proteins (DEPs)

**Fig. 8:**
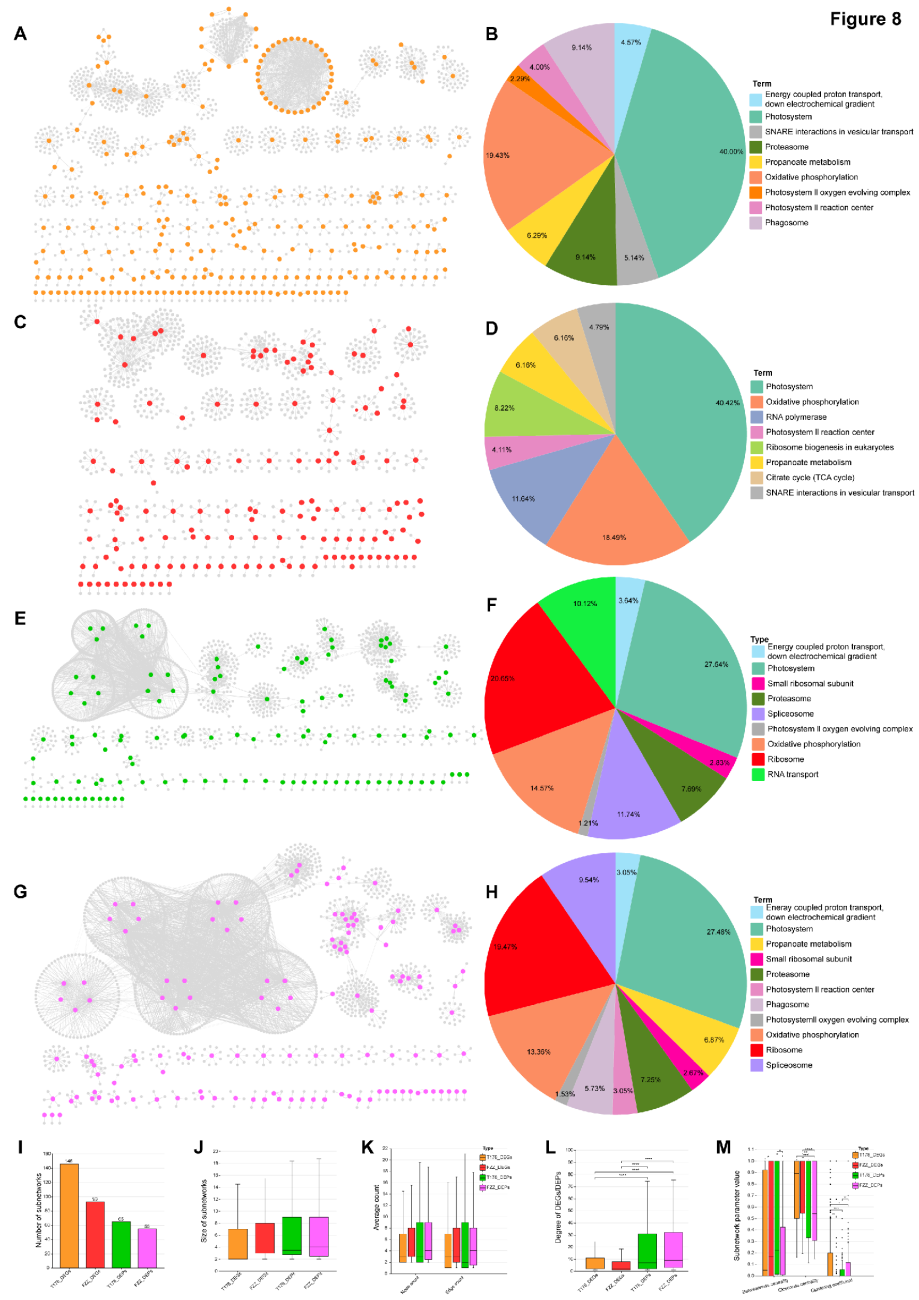
Comparative analysis of the topological structure and functional modules of PPI networks in FZZ and T178 under hypoxic conditions. (**A**) Protein-protein interaction network constructed based on DEGs in T178, where orange nodes represent DEGs in T178, and gray nodes represent proteins in the database that interact with T178’s DEGs. (**B**) Functional enrichment results of the PPI network for T178 DEGs, with different colored blocks representing different functional terms, and the size of the blocks indicating the proportion of each term. (**C**) PPI network constructed based on DEGs in FZZ, where red nodes represent DEGs in FZZ, and gray nodes represent proteins in the database that interact with FZZ’s DEGs. (**D**) Functional enrichment results of the PPI network for FZZ DEGs, with different colored blocks representing different functional terms, and the size of the blocks indicating the proportion of each term. (**E**) PPI network constructed based on differentially expressed proteins (DEPs) in T178, where green nodes represent DEPs in T178, and gray nodes represent proteins in the database that interact with T178’s DEPs. (**F**) Functional enrichment results of the PPI network for T178 DEPs, with different colored blocks representing different functional terms, and the size of the blocks indicating the proportion of each term. (**G**) PPI network constructed based on DEPs in FZZ, where purple nodes represent DEPs in FZZ, and gray nodes represent proteins in the database that interact with FZZ’s DEPs. (**H**) Functional enrichment results of the PPI network for FZZ DEPs, with different colored blocks representing different functional terms, and the size of the blocks indicating the proportion of each term. (**I**) Bar plot comparing the number of subnetworks in the PPI networks constructed from DEGs and DEPs of T178 and FZZ. (**J**) Boxplot comparing the size of subnetworks in the PPI networks constructed from DEGs and DEPs of T178 and FZZ. (**K**) Boxplot comparing the average number of nodes and edges in the subnetworks of the PPI networks constructed from DEGs and DEPs of T178 and FZZ. (**L**) Boxplot comparing the degree of DEG and DEP nodes in the PPI networks constructed from DEGs and DEPs of T178 and FZZ. (**M**) Boxplot comparing three network metrics - betweenness centrality, closeness centrality, and clustering coefficient - of the subnetworks in the PPI networks constructed from DEGs and DEPs of T178 and FZZ

To further elucidate the molecular response mechanisms of FZZ and T178 under hypoxic conditions, we constructed protein-protein interaction (PPI) networks based on differentially expressed genes (DEGs) and differentially expressed proteins (DEPs) for both (Fig. 8A, C, E, G). A systematic comparison of their hypoxia adaptation mechanisms was conducted from the perspectives of network topology and functional modules.

In terms of network topology analysis, the results are as follows: The PPI network of FZZ based on DEGs comprised 996 nodes (including 151 DEGs) and 1,193 edges, while its DEP network consisted of 1,312 nodes (including 133 DEPs) and 3,522 edges. In contrast, the DEG network of T178 was larger, containing 1,540 nodes (275 DEGs) and 2,547 edges, and its DEP network included 1,346 nodes (125 DEPs) and 2,684 edges. Further statistical analysis showed that the number of subnetworks in the four networks followed a decreasing trend: T178_DEGs > FZZ_DEGs > T178_DEPs > FZZ_DEPs (Fig. 8I), while the subnetwork size exhibited the opposite increasing order: T178_DEGs < FZZ_DEGs < T178_DEPs < FZZ_DEPs (Fig. 8J). By comparing the node count and edge count of the subnetworks (Fig. 8K), we found that the average node count per subnetwork was FZZ_DEPs > T178_DEPs > FZZ_DEGs > T178_DEGs, and the average edge count was FZZ_DEPs > T178_DEPs > T178_DEGs > FZZ_DEGs. Based on the aforementioned network topology characteristics, we hypothesize that T178 may reduce its sensitivity to hypoxic conditions by forming a greater number of relatively independent subnetwork modules. This decentralized regulatory mechanism enables T178 to coordinately respond to hypoxia through multiple independent modules. Even if the function of one module is compromised, other modules can maintain the system’s essential functions, thereby significantly enhancing overall stability. In contrast, FZZ exhibits fewer DEG subnetwork modules, with its gene regulation relying more heavily on a limited number of larger network modules. This centralized regulatory architecture renders the system more vulnerable to hypoxia, as the entire system’s functionality may be significantly compromised when key modules are affected by hypoxia.

Building upon the aforementioned analysis, we further investigated the degree values of differentially expressed genes (DEGs) and differentially expressed proteins (DEPs) nodes within the subnetworks. The results demonstrated that DEPs exhibited significantly higher degree values than DEGs (Fig. 8L), suggesting that the protein-level regulatory network may possess stronger functional integration capability. To comprehensively evaluate the functional roles and regulatory characteristics of DEGs and DEPs within the networks, we calculated three key network parameters for each node: Betweenness centrality, Closeness centrality, and Clustering coefficient. Specifically, Betweenness centrality measures the frequency at which a node appears on the shortest paths between all node pairs, reflecting its role as a “bridge” connecting different network modules; Closeness centrality measures the average shortest path length from a node to all other nodes in the network, indicating its centrality position within the network; and Clustering coefficient measures the degree of interconnectivity among a node’s neighbors, reflecting the local compactness of the network. The analytical results revealed the following patterns: Betweenness centrality followed T178_DEPs > FZZ_DEGs > T178_DEPs > FZZ_DEPs; Closeness centrality showed FZZ_DEGs > T178_DEGs > T178_DEPs > T178_DEPs; and Clustering coefficient demonstrated T178_DEGs > FZZ_DEPs > T178_DEPs > FZZ_DEGs (Fig. 8M). These findings provide further support for the hypothesis that T178 enhances its hypoxia tolerance through a decentralized, modular regulatory network, while simultaneously revealing significant differences in the gene- and protein-level regulatory networks between FZZ and T178.

To better understand the differences in hypoxia adaptation mechanisms between T178 and FZZ, we conducted functional enrichment analysis of the networks using the ClueGO plugin in Cytoscape. The results revealed that T178_DEGs were significantly enriched in pathways including photosynthesis (40.00%), oxidative phosphorylation (19.43%), proteasome (9.14%), and phagosome (9.14%). In comparison, while FZZ_DEGs showed similar enrichment levels in photosynthesis (40.42%) and oxidative phosphorylation (18.49%), they displayed specific enrichment in RNA polymerase (11.64%) and ribosome biogenesis (8.22%), and lacked the prominent protein degradation and immune response modules observed in T178. At the protein level, T178_DEPs exhibited higher enrichment proportions than FZZ_DEPs in ribosome (20.65% vs. 19.47%), spliceosome (11.74% vs. 9.54%), photosynthesis (27.54% vs. 27.48%), and oxidative phosphorylation (14.57% vs. 13.36%). Notably, FZZ_DEPs showed significantly lower enrichment in phagosome (5.73%) compared to T178_DEGs (9.14%, *p* < 0.05), indicating relatively weaker immune response capability (Fig. 8B, D, F, H). Based on these findings, we hypothesize that T178 has established a complex hypoxia adaptation network through multi-level coordinated regulation: (1) In energy metabolism, it coordinately regulates photosynthesis and oxidative phosphorylation; (2) In protein homeostasis, it integrates ribosome biogenesis with proteasomal degradation; (3) In RNA processing, it achieves post-transcriptional regulation through the spliceosome. In contrast, FZZ’s regulatory network exhibits distinct functional limitations: while its photosynthesis and ribosome function are comparable to T178, its enrichment proportions in crucial processes such as protein degradation, immune response, and RNA splicing are consistently lower than those of T178. This functional modular singularity and insufficient coordination may represent critical factors contributing to FZZ’s weaker hypoxia tolerance.

Finally, to obtain a more comprehensive perspective, we utilized the merge function in Cytoscape to integrate the four independent networks into a unified network (Supplementary Fig. S5). This consolidated network comprises 2,611 nodes and 8,357 edges. Statistical analysis revealed discrepancies in the number of differentially expressed genes (DEGs) and proteins (DEPs) between T178 and FZZ within the merged network. Specifically, T178 contributed 184 DEG nodes and 125 DEP nodes, while FZZ contributed only 77 DEG nodes and 65 DEP nodes. Additionally, the merged network contained 42 nodes shared by both species. Using cytoHubba, we identified the top 20 nodes with the highest degree values in the merged network, all of which were DEPs from T178 (Supplementary Table S28). This finding suggests that T178’s DEPs may serve as critical regulatory hubs, facilitating efficient stress response pathways under hypoxic conditions.

In conclusion, by constructing and analyzing protein-protein interaction (PPI) networks based on differentially expressed genes (DEGs) and differentially expressed proteins (DEPs) under hypoxic conditions, this study systematically compared the differences in hypoxia adaptation mechanisms between FZZ and T178. Network topology analysis revealed that T178 enhances its stability and tolerance under hypoxia by forming more relatively independent small network modules, whereas FZZ relies on fewer, larger network modules, making it more susceptible to hypoxia. Functional enrichment analysis further demonstrated that T178 exhibits stronger coordinated regulation across multiple critical biological processes, including photosynthesis, energy metabolism, protein synthesis and degradation, and RNA processing. In contrast, FZZ primarily depends on photosynthesis and ribosome function, lacking the prominent protein degradation and immune response modules observed in T178. These differences indicate that T178 enhances its hypoxia adaptation capability through a multi-level, multi-dimensional regulatory network, while the functional modular singularity and insufficient coordination in FZZ may be key factors contributing to its weaker hypoxia tolerance.

## Discussion

Under flooding conditions, plant roots are the primary organ to suffer from hypoxia. Our previous study revealed that hypoxia inhibits root growth; however, this inhibition is less pronounced in wild tomato T178 compared to cultivated tomato FZZ [30]. In this study, we also found that hypoxia decreased dry weight in FZZ more than that in T178 (Fig. 1). Further research is needed to elucidate the molecular mechanisms responsible for the differing hypoxia tolerance between wild and cultivated tomatoes.

Plant hormones are essential signaling molecules that orchestrate the regulation of gene expression under hypoxia [52]. A recent study has unveiled the impact of flooding on the synthesis and transport of plant hormones. This investigation demonstrated the suppression of cytokinin (CTK) and gibberellin (GA) synthesis in roots, as well as the inhibition of ethylene (ETH), abscisic acid (ABA), and indole-3-acetic acid (IAA) transport in shoots. Consequently, the accumulation of these hormones in shoots leads to premature senescence [53]. In this study, the imposition of hypoxia resulted in significant alterations in the expression of four differentially expressed genes (DEGs) associated with ethylene synthesis and metabolic pathways (Fig. 7B). Fukao et al. (2008) found that Sub1A, encoding ethylene response factors (*ERFs*), promotes ethylene synthesis, with ethylene-inducing ABA degradation, and inhibits the GA response to regulate the response to flooding and hypoxia [54]. *AP2/ERF* plays an important role in the hypoxia response. Research revealed that *ERFs* are involved in the accumulation of ethylene, CTK-mediated cell senescence, and GA-mediated stem elongation [55]. Our results found that hypoxia stress significantly increases the expression of ETR, *EBF1/2*, and *ERF1* (Fig. 7B). Ethylene promotes auxin synthesis and accumulation in root tips of *Arabidopsis* [56, 57], but ethylene negatively regulates IAA content in tomato roots [58]. A study has demonstrated the essential role of ethylene in promoting an increase in auxin content in tomatoes subjected to waterlogging conditions [26]. Upon exposure to hypoxia, *Arabidopsis* exhibits a rapid increase in the transcriptional levels of auxin response genes *IAA2* and *IAA3* [59]. In our study, the application of hypoxia stress resulted in significantly higher expression of auxin-related genes (*AUX/IAA* and *SAUR*) in both T178 and FZZ tomato genotypes. Collectively, these findings demonstrate the crucial roles played by certain ethylene- and auxin-related genes in conferring tolerance to hypoxia in both wild and cultivated tomato varieties.

The regulation of gene expression pertaining to primary metabolism and energy homeostasis plays a crucial role in mitigating energy crisis under hypoxic conditions [60]. Studies conducted on rice have demonstrated that both flooding and hypoxia conditions lead to the inhibition of aboveground elongation and reduction in carbohydrate consumption [54] and that the transcript level of GAPDH (*LOC_Os02g38920*), which is related to glycolysis, was significantly increased in the hypoxia-tolerant cultivar YF [5]. In this experiment, the expression of the gene *GAPDH* (*Solyc12g094640*) was also significantly higher in the hypoxia-treated variety T178. In contrast, *GAPDH* gene expression in FZZ was decreased. Hypoxia treatment significantly induced the expression of genes related to glycolysis and carbon metabolism pathways in both T178 and FZZ (Supplementary Table S18). The differences in expression of the related genes in the T178-N group were more significant, especially for the *Solyc02g077240* and *Solyc10g076510* genes in the glycolytic pathway, which were differentially up-regulated at a significantly increased multiplicity than that of FZZ-N. Following exposure to flooding stress, plants experienced inhibition of aerobic respiration within the root system, resulting in the blockade of ATP synthesis. Consequently, the produced energy became inadequate to sustain the diverse physiological, biochemical, and metabolic activities within the plant [16]. Under hypoxic conditions, a significant decline in the content of carbohydrates and soluble sugars was observed in rice and wheat, coinciding with the apparent deterioration and eventual demise of the plants [61]. Carbohydrates serve as the principal energy storage compounds in plants and are closely associated with the plants’ hypoxia tolerance. Under hypoxia conditions, the activation of metabolic pathways, such as glycolysis and fermentation, represents the primary mechanism by which plants acquire energy replenishment [62]. Based on our findings, it can be inferred that disparities in the expression of genes associated with carbon metabolism and glycolysis between the two tomato species may serve as a crucial determinant for the heightened hypoxia tolerance observed in the wild tomato genotype T178 in comparison to FZZ.

The *CNGC* (cyclic nucleotide-gated channel) has a wide range of functions in the control of ionic homeostasis, development, and defense against biotic and abiotic stresses [63]. *WRKY* (WRKY DNA-binding protein) transcription factors are emerging as the most typical class of plant transcription factors and are at the forefront of research on plant defense responses [64, 65]. Interestingly, hypoxia treatment resulted in significant down-regulation of gene expression related to phytopathogen pathways in both FZZ and T178. Nevertheless, the wild species exhibited higher expression levels of *CNGC*, *CML* (calcium-binding protein), and *WRKY* genes in comparison to FZZ (Supplementary Table S19). These genes are involved in the pathway of plant-pathogen interactions and are differentially expressed in the two tomato species, and may be the significant regulatory genes for hypoxia stress resistance in cultivated tomato FZZ.

Domestication has historically served as a prominent paradigm illustrating the substantial phenotypic variations arising from selective pressures [66, 67]. Studies conducted on various species, including maize and rice, have provided compelling evidence supporting the notion that the genomes across these organisms possess a minor fraction of genes exhibiting evidence of positive selection during the process of domestication [68, 69]. Our investigation revealed the presence of genetic signatures indicating positive selection in specific genes within both the wild tomato genotype T178 and the cultivated tomato genotype FZZ. Notably, these selected genes were primarily enriched in functional pathways related to tryptophan metabolism, protein processing in the endoplasmic reticulum, and plant-pathogen interactions. In terms of tryptophan metabolism, it has been shown that tryptophan is a pre-material for plant defense compound indole glucosinolates [70], whereas protein processing in endoplasmic reticulum may be related to plant stress under environmental stress [71]. The favorable selection observed in tomatoes during the course of their environmental history, in response to environmental stresses and microbial disease pressures, is aligned with the agricultural requirements of human cultivation production needs. The fact that the overlapping gene set showed signs of stronger positive selection in the cultivated genotype FZZ, in comparison to the wild tomato genotype T178, implies that the cultivated tomato FZZ has undergone greater domestication influences.

## Conclusions

By integrating RNA-seq and proteomic analyses, this study elucidates the molecular mechanisms underlying the divergent hypoxia tolerance observed in wild (T178) and cultivated (FZZ) tomatoes. Hypoxic stress significantly altered the expression of genes involved in plant hormone signaling pathways, particularly those related to ethylene response factors (ERFs) and auxin metabolism, indicating their crucial role in conferring hypoxia tolerance. Both T178 and FZZ showed enrichment in carbon and energy metabolism pathways, yet differential expression patterns suggest that T178 more effectively maintains energy homeostasis under low-oxygen conditions. Additionally, T178 exhibited higher expression levels of genes associated with pathogen interaction pathways than FZZ. Proteomic data further supported these transcriptomic findings, highlighting the importance of energy-related processes in hypoxia adaptation. PPI network analyses revealed that FZZ’s DEP-based networks are more complex, emphasizing the critical role of differentially expressed proteins (DEPs) under hypoxia, while T178’s DEG-based networks display greater flexibility, potentially enhancing its ability to withstand low-oxygen stress. Collectively, these results identify key genes and pathways-particularly those related to protein synthesis, photosynthesis, and antioxidant responses-that may be harnessed to improve hypoxia tolerance. This work provides a theoretical foundation for breeding strategies aimed at developing tomato cultivars better adapted to hypoxic environments.

## Electronic supplementary material

Below is the link to the electronic supplementary material.

Supplementary Material 1

Supplementary Material 2

Supplementary Material 3

Supplementary Material 4

Supplementary Material 5

Supplementary Material 6

Supplementary Material 7

Supplementary Material 8

## Data Availability

The paired-end RNA-seq data from our study on the comparative analysis of root transcriptomes in wild and cultivated tomatoes under hypoxia conditions have been submitted NCBI SRA database under the BioProject accession number PRJNA951445. The associated BioSamples accessions and library IDs are detailed as follows: In wild tomato (T178), the BioSamples for the hypoxia treatment were SAMN34042777 (Library ID: SRR24044094, T178-N1) and SAMN34042778 (Library ID: SRR24044093, T178-N2). For the aeration treatment, the BioSamples were SAMN34042776 (Library ID: SRR24044095, T178-A2) and SAMN34042775 (Library ID: SRR24044096, T178-A1). In addition, in cultivated tomato (FZZ), hypoxia-treated BioSamples were SAMN34042773 (Library ID: SRR24044098, FZZ-N1) and SAMN34042774 (Library ID: SRR24044097, FZZ-N2), respectively. Under aerated treatment conditions, the biological samples were SAMN34042772 (Library ID: SRR24044099, FZZ-A2) and SAMN34042771 (Library ID: SRR24044100, FZZ-A1). All sequence files are available in FASTQ format with pair-end reads.

## Abbreviations

DEGs: Differentially expressed genes
DEPs: Differentially expressed proteins
ROS: Reactive oxygen species
LC: liquid chromatography
CDS: Coding sequences
GO: Gene ontology
KEGG: Kyoto encyclopedia of genes and genomes
T178: Solanum habrochaites
FZZ: Solanum lycopersicum
PPI: Protein-protein interaction
ERFs: Ethylene response factors
