# Supplementary figures and images for: Comparative transcriptomic and proteomic analyses of hypoxia response in wild and cultivated tomato roots
Source: BMC Genomics. 2025 Jun 2;26:552. doi: 10.1186/s12864-025-11653-3 (PMC12128530; doi:10.1186/s12864-025-11653-3)

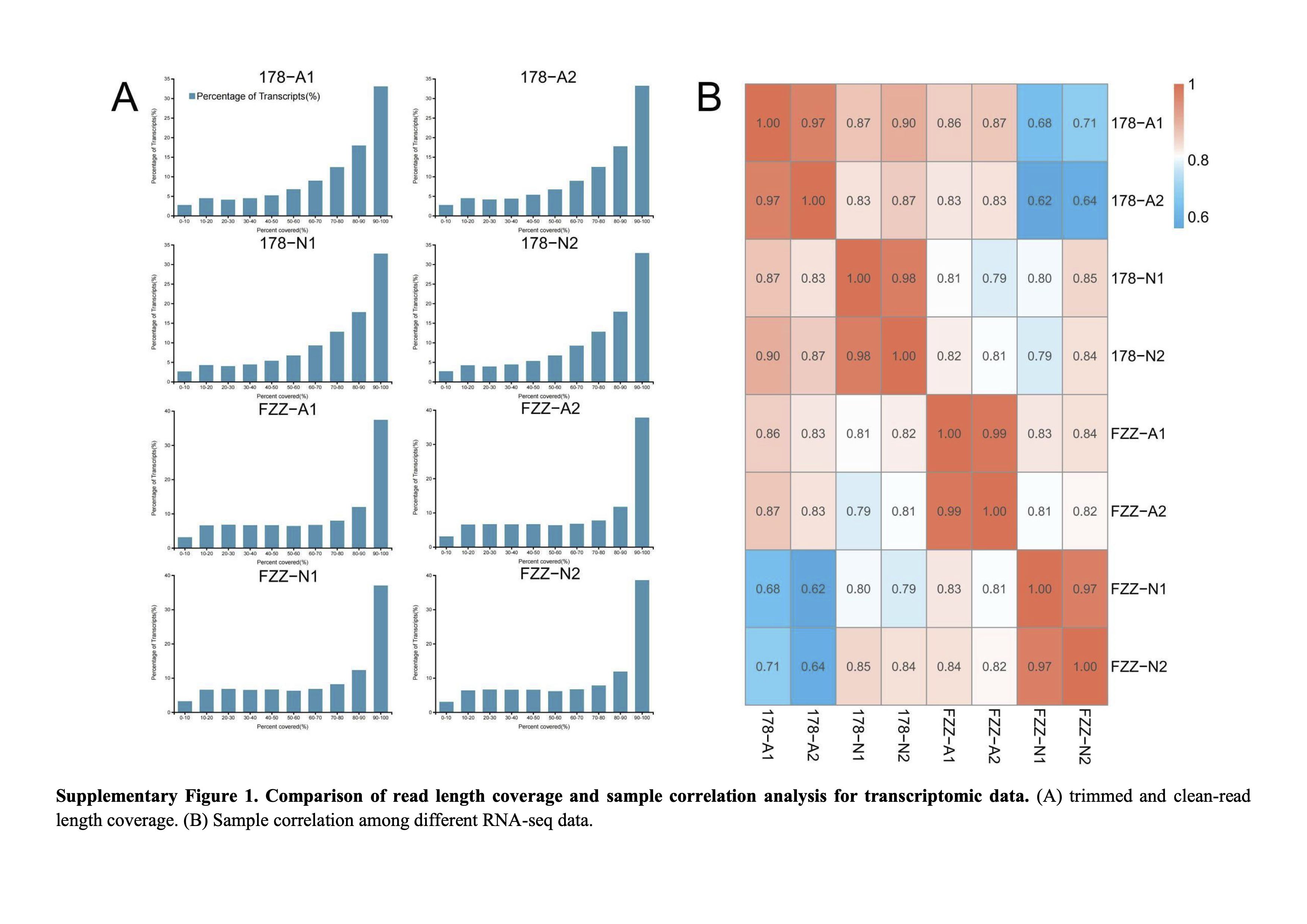

Supplement: Supplementary file 2 — Supplementary Material 2 [file 12864_2025_11653_MOESM2_ESM.tif]

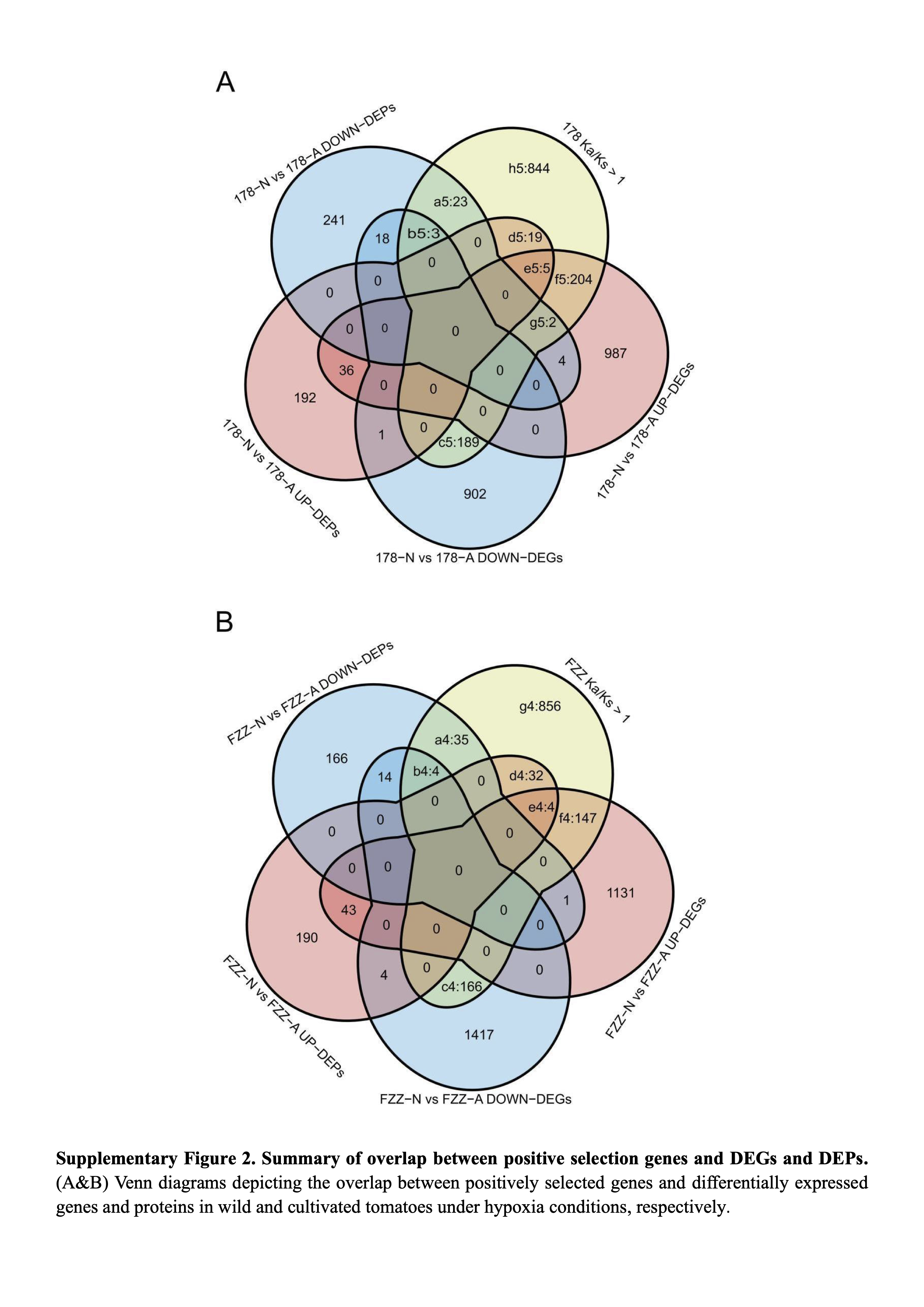

Supplement: Supplementary file 3 — Supplementary Material 3 [file 12864_2025_11653_MOESM3_ESM.tif]

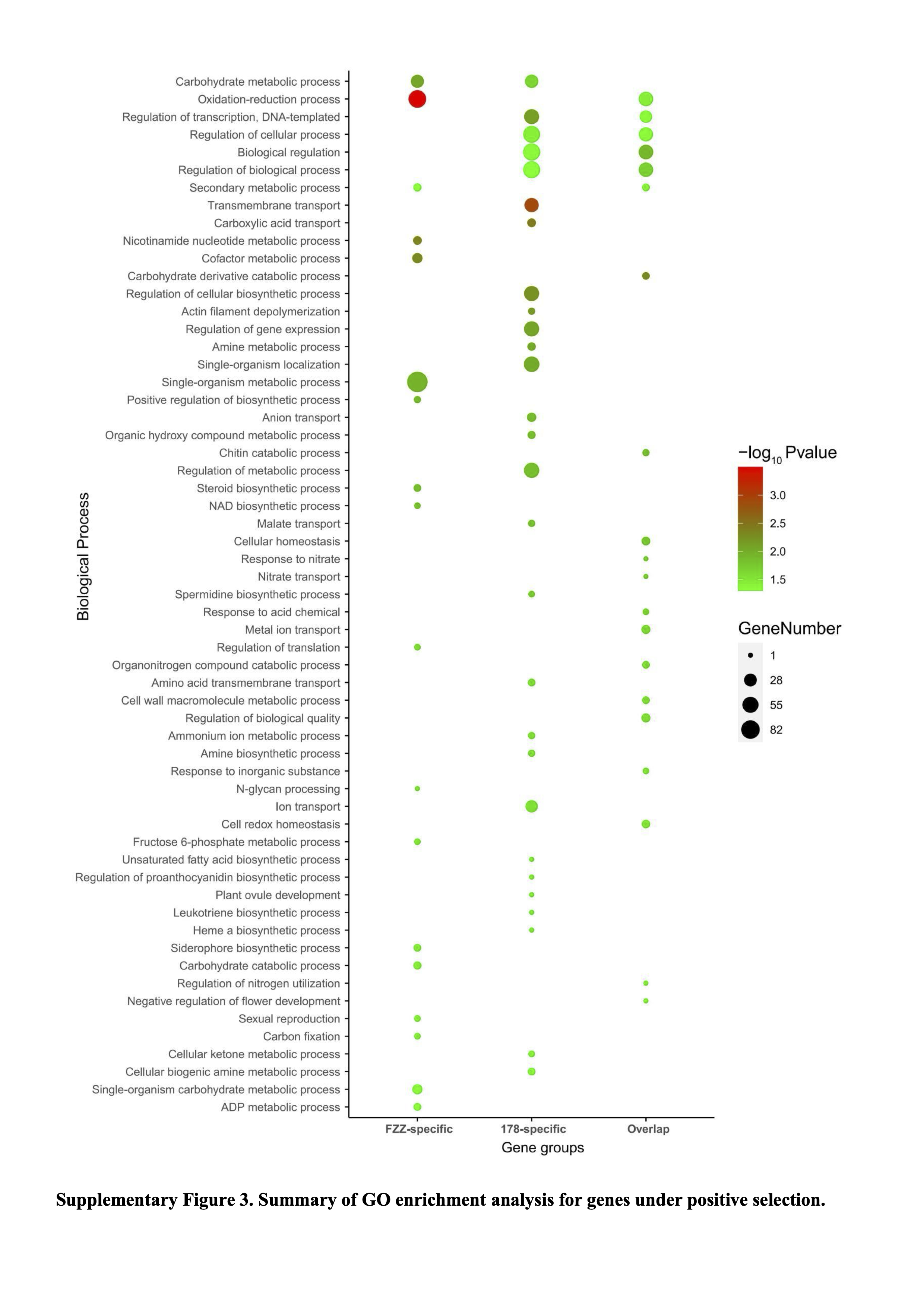

Supplement: Supplementary file 4 — Supplementary Material 4 [file 12864_2025_11653_MOESM4_ESM.tif]

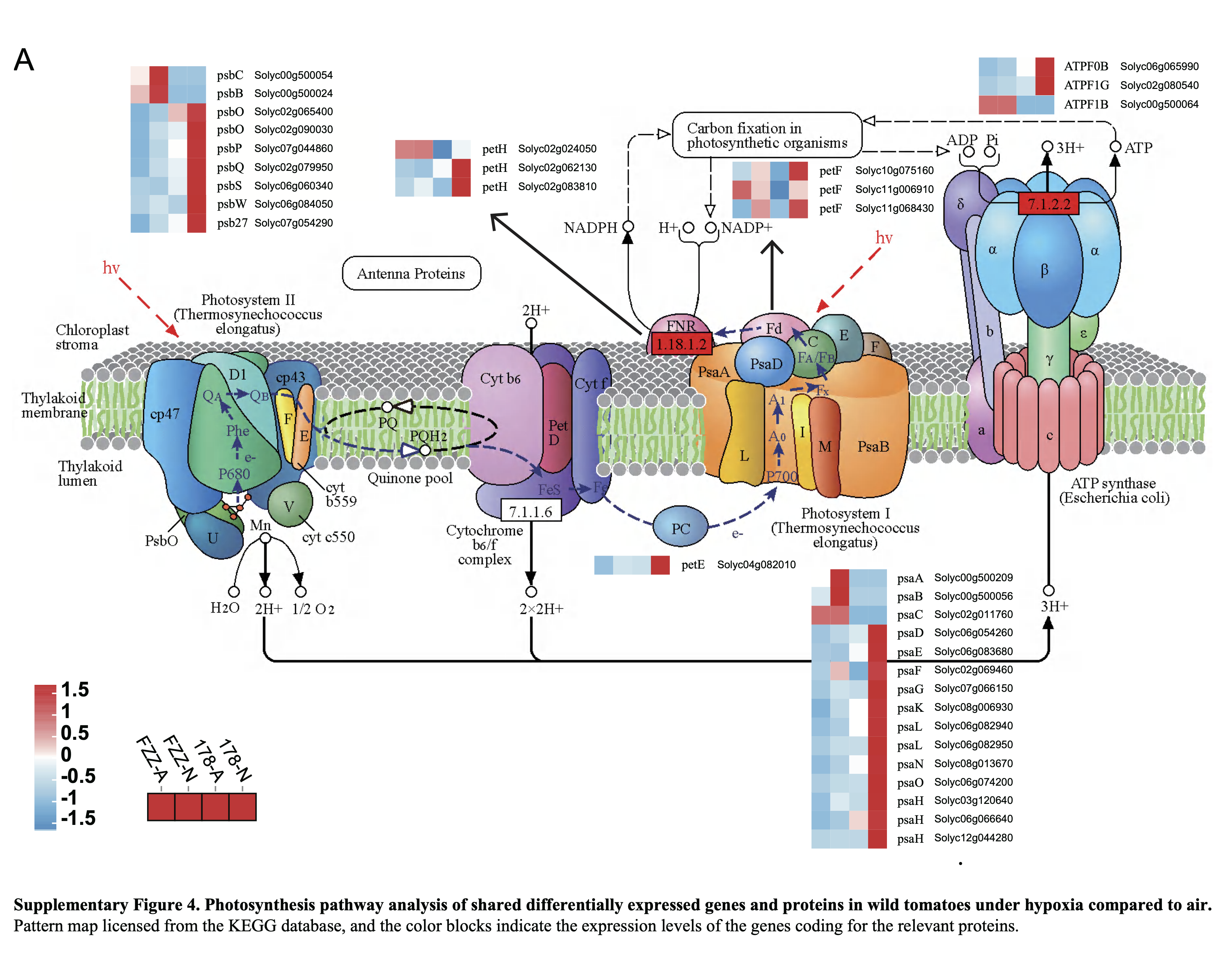

Supplement: Supplementary file 5 — Supplementary Material 5 [file 12864_2025_11653_MOESM5_ESM.tif]

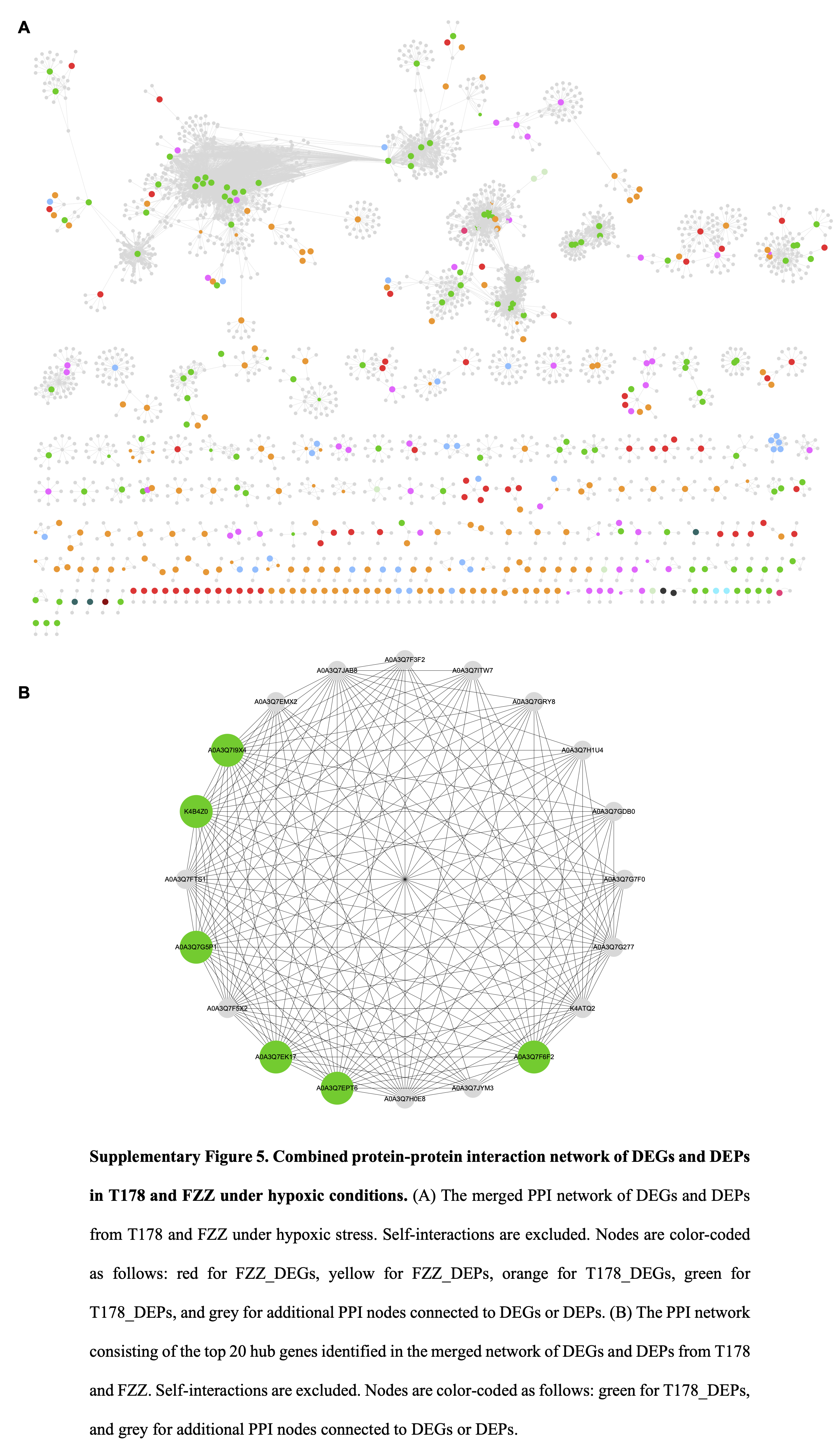

Supplement: Supplementary file 6 — Supplementary Material 6 [file 12864_2025_11653_MOESM6_ESM.tif]

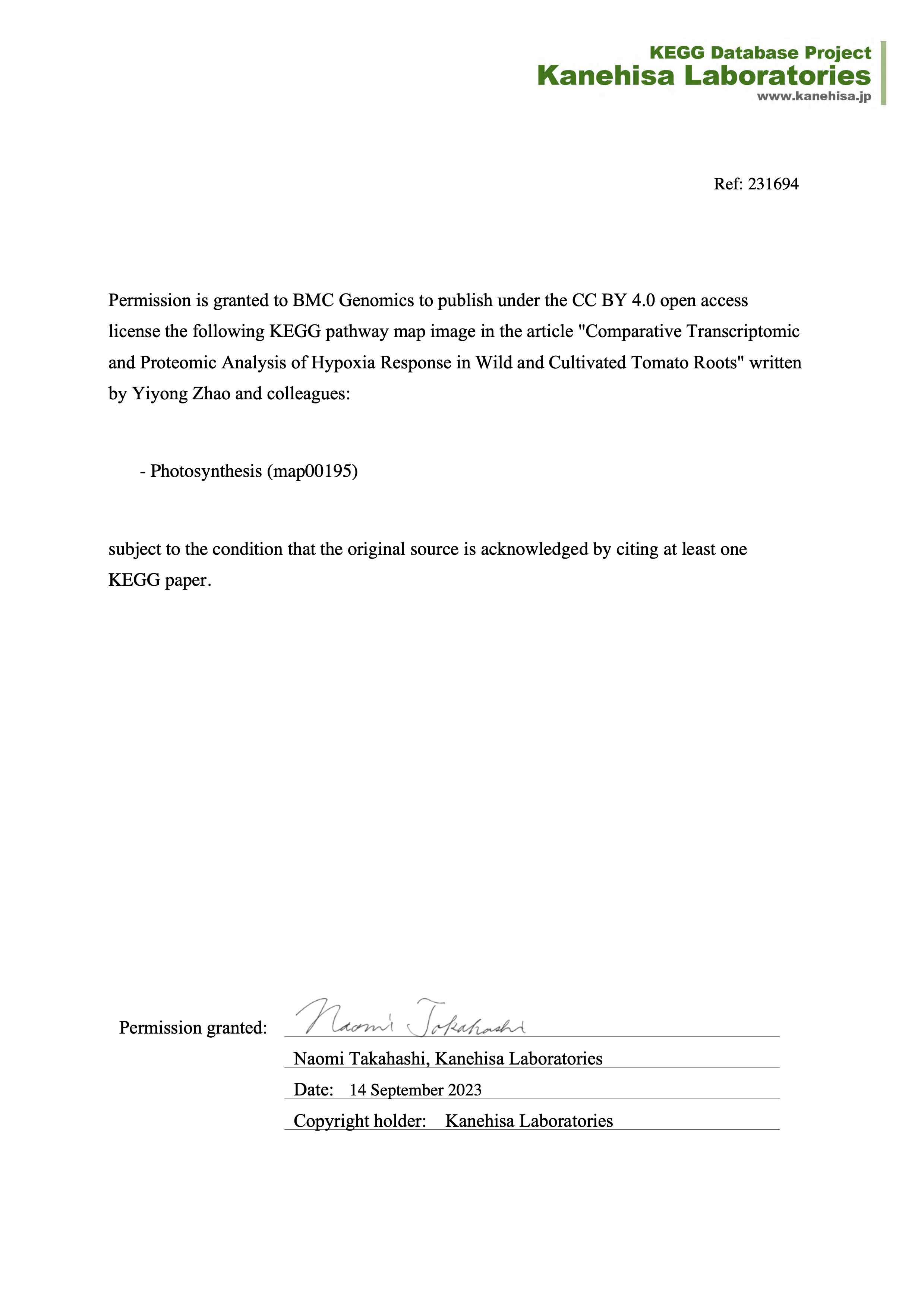

Supplement: Supplementary file 7 — Supplementary Material 7 [file 12864_2025_11653_MOESM7_ESM.tif]
