# Supplementary material for: Comparative transcriptomic and proteomic analyses of hypoxia response in wild and cultivated tomato roots
Source: BMC Genomics. 2025 Jun 2;26:552. doi: 10.1186/s12864-025-11653-3 (PMC12128530; doi:10.1186/s12864-025-11653-3)
Supplement: Supplementary file 8 — Supplementary Material 8 [file 12864_2025_11653_MOESM8_ESM.docx]

**Supplementary Information:**

**Supplementary Figure legend:**

**Supplementary Fig. 1.** Comparison of read length coverage and sample correlation analysis for transcriptomic data. A. trimmed and clean-read length coverage. B. Sample correlation among different RNA-seq data.

**Supplementary Fig. 2.** Summary of overlap between positive selection genes and DEGs and DEPs. (A&B) Venn diagrams depicting the overlap between positively selected genes and differentially expressed genes and proteins in wild and cultivated tomatoes under hypoxia conditions, respectively.

**Supplementary Fig. 3.** Summary of GO enrichment analysis for genes under positive selection.

**Supplementary Fig. 4.** Photosynthesis pathway analysis of shared differentially expressed genes and proteins in wild tomatoes under hypoxia compared to air. Pattern map licensed from the KEGG database, and the color blocks indicate the expression levels of the genes coding for the relevant proteins.

**Supplementary Fig. 5.** Combined protein-protein interaction network of DEGs and DEPs in T178 and FZZ under hypoxic conditions. (A) The merged PPI network of DEGs and DEPs from T178 and FZZ under hypoxia. Self-interactions are excluded. Nodes are color-coded as follows: red for FZZ_DEGs, yellow for FZZ_DEPs, orange for T178_DEGs, green for T178_DEPs, and grey for additional PPI nodes connected to DEGs or DEPs. (B) The PPI network consisting of the top 20 hub genes identified in the merged network of DEGs and DEPs from T178 and FZZ. Self-interactions are excluded. Nodes are color-coded as follows: green for T178_DEPs, and grey for additional PPI nodes connected to DEGs or DEPs.

**Supplementary Fig. 6.** Authorization document for Supplementary Fig. 4.

**Supplementary Table legend:**

**Supplementary Table 1.** Impact of hypoxia and normoxia treatments on the growth of FZZ and T178 tomato seedlings. "±" represents the standard error (SE), indicating the variability around the mean. A smaller standard error suggests higher reliability of the measurements. The letters following the data (a, b) indicate significant differences. When multiple groups are compared using statistical methods (Tukey multiple comparison test), different letters signify significant differences between groups.

**Supplementary Table 2.** Membership function values and sensitivity evaluation of tomato seedlings under hypoxic and normoxic treatments. Membership function value ≥ 0.7: Highly sensitive, classified as level I. Membership function value ≥ 0.6: Sensitive, classified as level II. Membership function value ≥ 0.4: Moderately sensitive, classified as level III. Membership function value ≥ 0.3: Weakly sensitive, classified as level IV. Membership function value < 0.3: Insensitive, classified as level V.

**Supplementary Table 3.** Overview of sequencing and read mapping statistics for the tomato transcriptomes.

**Supplementary Table 4.** Differential gene expression analysis of *Solanum habrochaites* T178 under hypoxia compared to normoxic conditions from RNA-seq data. Table lists normalized CPM (Counts Per Million) and TPM (Transcripts Per Million) value derived using the DEseq2 and read counts as quantified by StringTie.

**Supplementary Table 5.** Differential gene expression analysis of *Solanum lycopersicum* FZZ under hypoxia compared to normoxic conditions from RNA-seq data. Table lists normalized CPM (Counts Per Million) and TPM (Transcripts Per Million) value derived using the DEseq2 and read counts as quantified by StringTie.

**Supplementary Table 6.** Hypoxia-responsive genes from *Arabidopsis*, identified as significantly differentially expressed in response to hypoxia, as revealed by the tomato transcriptome analysis conducted in this study.

**Supplementary Table 7.** GO enrichment analysis for differentially expressed genes derived from the transcriptomic data.

**Supplementary Table 8.** KEGG enrichment analysis for differentially expressed genes derived from the transcriptomic data.

**Supplementary Table 9.** Differential protein expression analysis and annotation of *S. habrochaites* T178 under hypoxia compared to normoxic conditions from proteomic data. The description of abbreviations of each column name is attached at the end of the table. The description of abbreviations of each column name is attached at the end of the table.

**Supplementary Table 10.** Differential protein expression analysis and annotation of *Solanum lycopersicum* FZZ under hypoxia compared to normoxic conditions from proteomic data.

**Supplementary Table 11.** GO enrichment analysis for differentially expressed proteins identified from the proteomic data.

**Supplementary Table 12.** KEGG enrichment analysis for differentially expressed proteins derived from the transcriptomic data.

**Supplementary Table 13.** Comprehensive summary of combined transcriptomic and proteomic GO enrichment analysis.

**Supplementary Table 14.** KEGG enrichment analysis of overlapping genes for DEPs and DEGs derived from transcriptome data.

**Supplementary Table 15.** Positive selection analysis for hypoxia-responsive related genes in both cultivated and wild tomatoes.

**Supplementary Table 16.** Rapidly upregulated key genes in plant hormone signal transduction pathway under hypoxia in both cultivated and wild tomatoes.

**Supplementary Table 17.** Key photosynthesis pathway genes exhibiting differential regulation under hypoxia across both cultivated and wild tomatoes.

**Supplementary Table 18.** Glycolysis / gluconeogenesis and carbon metabolism pathway genes exhibiting differential regulation under hypoxia across both cultivated and wild tomatoes.

**Supplementary Table 19.** Plant-pathogen interaction pathway genes exhibiting differential regulation under hypoxia across both cultivated and wild tomatoes.

**Supplementary Table 20.** Correspondence of gene IDs for T178 differentially expressed genes in the STRING PPI network.

**Supplementary Table 21.** PPI network information for DEGs of T178.

**Supplementary Table 22.** Correspondence of gene ids for T178 differentially expressed proteins in the STRING PPI network.

**Supplementary Table 23.** PPI network information for DEPs of T178.

**Supplementary Table 24.** Correspondence of gene IDs for FZZ differentially expressed genes in the STRING PPI network.

**Supplementary Table 25.** PPI network information for DEGs of FZZ.

**Supplementary Table 26.** Correspondence of gene IDs for FZZ differentially expressed proteins in the STRING PPI network.

**Supplementary Table 27.** PPI network information for DEPs of FZZ.

**Supplementary Table 28.** Top 20 nodes with highest degree in the merged PPI network.
